# Microbiome-mediated polyphosphate accumulation enhances the resilience of sponge holobionts to future climate scenarios

**DOI:** 10.1093/ismejo/wrag128

**Published:** 2026-05-29

**Authors:** Bifu Gan, Chenyu Yang, Chenzheng Jia, Kai Wang, Jun Chen, Shaoxiong Ding, Jing Zhao

**Affiliations:** State Key Laboratory of Marine Environmental Science, College of Ocean and Earth Sciences, Xiamen University, Xiamen 361000, Fujian Province, China; State Key Laboratory of Mariculture Breeding, College of Ocean and Earth Sciences, Xiamen University, Xiamen 361000, Fujian Province, China; Fujian Provincial Key Lab of Coastal Basin Environment, Fujian Polytechnic Normal Univeristy, Fuqing 350300, Fujian Province, China; State Key Laboratory of Mariculture Breeding, College of Ocean and Earth Sciences, Xiamen University, Xiamen 361000, Fujian Province, China; Fujian Ocean Innovation Center, Xiamen 361102, Fujian Province, China; State Key Laboratory of Mariculture Breeding, College of Ocean and Earth Sciences, Xiamen University, Xiamen 361000, Fujian Province, China; Fujian Ocean Innovation Center, Xiamen 361102, Fujian Province, China; State Key Laboratory of Mariculture Breeding, College of Ocean and Earth Sciences, Xiamen University, Xiamen 361000, Fujian Province, China; Fujian Ocean Innovation Center, Xiamen 361102, Fujian Province, China; State Key Laboratory of Mariculture Breeding, College of Ocean and Earth Sciences, Xiamen University, Xiamen 361000, Fujian Province, China; State Key Laboratory of Marine Environmental Science, College of Ocean and Earth Sciences, Xiamen University, Xiamen 361000, Fujian Province, China; State Key Laboratory of Mariculture Breeding, College of Ocean and Earth Sciences, Xiamen University, Xiamen 361000, Fujian Province, China; Fujian Ocean Innovation Center, Xiamen 361102, Fujian Province, China; State Key Laboratory of Mariculture Breeding, College of Ocean and Earth Sciences, Xiamen University, Xiamen 361000, Fujian Province, China; Fujian Ocean Innovation Center, Xiamen 361102, Fujian Province, China

**Keywords:** polyphosphates, *ppk1*-harboring microbial assemblages, ocean warming, sponge holobionts, future climatic conditions, sponge-associated microorganisms, sponge host

## Abstract

Ocean warming is threatening the stability of marine ecosystems, yet the mechanisms underlying the resilience of foundational species like sponges remain poorly understood. Polyphosphate (PolyP), a key player in marine phosphorus burial, is hypothesized to play an important role in sponge stress adaptation. Here, we demonstrated that microbiome-mediated PolyP dynamics were closely associated with sponge adaptation to thermal stress. Field investigation of three sponge species (*Spongia* sp*., Tedania* sp*., Haliclona simulans*) revealed marked interspecific differences in PolyP accumulation, with *Spongia* sp. maintaining the highest PolyP levels. Strong seasonal fluctuations in sponge PolyP content, peaking in June and reaching a minimum in January, correlated with ambient temperature. We found that this variation was regulated by *ppk1* gene expression levels rather than by altering the microbial composition. The *ppk1*-harboring microbial assemblages exhibited host specificity, and phylogenetic analysis uncovered sponge-specific clades of *ppk1* genes. Laboratory warming experiments further confirmed the functional link: under acute heat stress, both the *ppk1* gene of the symbiotic microorganisms and the inorganic pyrophosphatase (*ppa*) gene in the host were upregulated in *Spongia* sp. PolyP likely provide an energy source for the host, maintaining holobiont stability and leading to low mortality. Conversely, *H. simulans*, with limited PolyP supply, ultimately suffered 100% mortality. Our results establish a link whereby sponge-associated microbes, via *ppk1* expression, modulate PolyP accumulation to support host oxidative phosphorylation and mitigate thermal stress. This microbiome-mediated physiological pathway contributes to our understanding of sponge climate resilience, offering new functional insights into holobiont persistence in a warming ocean.

## Introduction

The inevitable continuation of the global ocean surface temperature rise will impact many biological processes [1], thereby disrupting population dynamics and ecosystem functioning [2]. Warming oceans have driven yearly declines in coral cover [3–5], potentially altering benthic reef community structure [6]. Meta-analysis predicted that certain sponges possess a greater adaptive capacity to climate change than other benthic organisms [7–9]. As key members of coral reefs, sponges host highly complex and diverse microbial communities, forming functionally important “sponge holobionts” [10] that contribute significantly to habitat formation, nutrient cycling, and biological erosion [10–12].

Sponge holobionts significantly affect the cycling of carbon, nitrogen, and phosphorus. Fossil evidence suggests that sponges facilitated phosphate mineralization as early as 480 million years ago [13]. They can enrich phosphorus and synthesize polyphosphate (PolyP), playing an important role in marine phosphorus burial [14–16]. PolyP, a ubiquitous linear–linear polymer of phosphate residues [17], serves diverse cellular functions, including phosphate and energy storage, membrane transport, cell envelope function, enzyme activity regulation, and homeostasis adaptation [17–20]. It is essential for cell growth, stress response, and defense against pathogens [17]. However, PolyP research has primarily focused on free-living bacteria or phytoplankton, largely neglecting its study in other marine organisms [14, 21].

Sponges have significant phosphorus-accumulating capabilities, which can be attributed to their symbiotic microorganisms. Sponge-associated bacteria synthesizing PolyP may be among the earliest bacteria to directly store nutrients for the host [22]. Various bacterial lineages, such as *Pseudomonodota, Cyanobacteriota, Actinomycetota*, and *Acidobacteriota*, are potentially involved in dissolved inorganic phosphate (DIP) accumulation within the host [15]. Key PolyP synthesis enzymes, particularly PolyP kinase 1 encoded by the *ppk1* genes, have been identified in sponge symbionts [14, 15, 23]. However, the limited scope of *ppk1* gene studies restricts our understanding of its prevalence and function across the broader sponge symbiotic microbiome.

Prior research shows that specific sponge-associated bacteria accumulate PolyP under oligotrophic conditions, highlighting a conserved nutrient storage strategy [14, 15]. In the freshwater sponge *Ephydatia muelleri*, PolyP level decreases significantly during germination and hatching, but increases upon exposure to polluted river water [24]. Furthermore, PolyP accumulation in some organisms (e.g. picoplankton) changes markedly with increasing temperature [25], and environmental variation leads to significant differences in PolyP content within the same sponge species [15]. These findings imply that PolyP plays a potential role in coordinating sponge physiology and environmental stress, including thermal stress. Changes in PolyP content may reflect the holobiont’s adaptive response. We propose that PolyP could be important for sponge host adaptability and stress recovery under climate change, but the specific mechanism and ecological significance remain unclear.

Some sponge species exhibit resilience to thermal stress [7]. The sponge *Neopetrosia compacta* maintains a stable microbiome and host transcriptome despite temperature fluctuations [26]. Similarly, heat stress has no significant effect on the survival of several Caribbean sponges [27] or the deep-sea sponge *Geodia barretti* [28]. Conversely, elevated temperature can cause tissue necrosis in other species such as *Stylissa flabelliformis*, and alter microbial community composition, weaken host–microbe interactions, and impair nutrient cycling [29]. Although stress-induced disruptions to host–microbe interactions are common, the plasticity of the microbiome provides potential pathways for under environmental threats [30, 31].

This study focuses on microbially synthesized PolyP in sponge responses to ocean warming, integrating field investigations with laboratory experiments that simulate future marine conditions. Specifically, we aim to: (i) determine how PolyP accumulation in sponge changes under temperature stress; (ii) analyze the reasons driving these changes; and (iii) elucidate how PolyP aids the holobiont’s stress response. This work investigates the potential role of PolyP in the response of sponges to ocean warming. Understanding these host–microbe interactions is crucial for assessing sponge adaptive potential and predicting shifts in their ecological distribution under future climates.

## Materials and methods

### Sampling and environmental factor measurements

Three sponge species, *Spongia* sp., *Tedania* sp., and *Haliclona simulans*, were sampled at Zhao’an Bay, Zhangzhou City, Fujian Province, China (at 23.62°N, 117.34°E, ambient temperature: 16°C–30°C [32]) in 2022. Sponges were collected in triplicate each month for 1 year. All samples (~3 cm^3^ each) were collected from the same marked individuals throughout the year, with each sampling removing an estimated 1/30 to 1/15 of the individual’s volume. Individual surfaces of all sponge samples were rinsed with filtered natural seawater (0.22 μm filter, Millipore, Billerica, MA, USA) and divided into three pieces. One piece was immersed in 70% (vol/vol) ethanol, temporarily stored on ice during transport, and subsequently stored at −80°C upon arrival in the laboratory. The second was immersed in RNAlater (Invitrogen), placed at 4°C overnight, then stored at −80°C until use. The third was processed for PolyP granules visualization following the previously described method [14]. Seawater physicochemical parameters were measured as described previously [32].

### Extraction, measurement and visualization of total polyphosphate from sponge tissue

**Figure 1:**
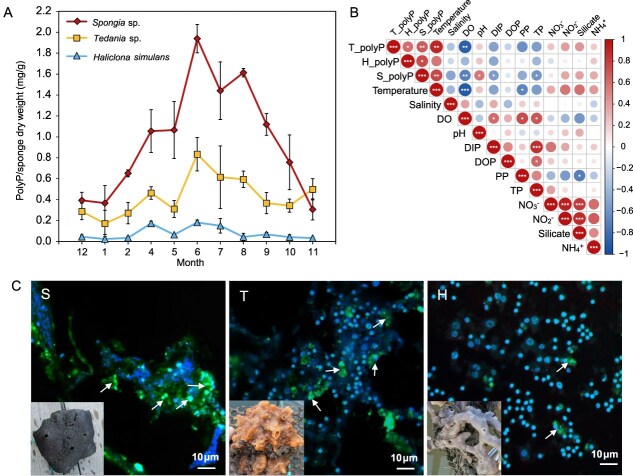
PolyP levels in different sponge species and its correlation with the environmental factors. (A) Monthly variation of PolyP levels in sponges. (B) Correlation between PolyP levels and environmental factors. (C) Visualization of PolyP in sponge tissues by fluorescence microscopy. The particles indicated by the arrow are high-concentration PolyP particles. Significances levels: ^*^: *P* < .05, ^**^: *P* < .01, ^***^: *P* < .001. S = *Spongia* sp., T = *Tedania* sp., H = *H. simulans*.

Total PolyP was extracted and quantified from sponge tissue following previously described method [14]. For each species, tissue from three replicates individuals underwent five consecutive extractions (Figs S1 and S2). To evaluate the capacity of the sponge to accumulate PolyP, the enrichment rate was defined as the ratio of PolyP in the sponge tissue to DIP in the surrounding water. PolyP granules were visualized under a Zeiss LSM780NLO (Carl Zeiss AG, 73447, Oberkochen, Germany) inverted confocal microscope (Figure  1). Detailed methods are in Supplementary Materials 2.

### Primer design and validation for *ppk1* gene

Full-length prokaryotic *ppk1* nucleotide sequences were retrieved from the National Center for Biotechnology Information (NCBI). After translating and comparing the deduced amino acid sequence, primers were designed. Based on the results of the gradient PCR trials, primers ppk1F and ppk1R (Table 1, Fig. S3) as optimal, amplifying a 578 bp fragment. Primers for quantitative real-time PCR (qRT-PCR) of *ppk1* and the eukaryotic *vtc4* gene (involved in PolyP synthesis in organisms such as fungi and algae) were similarly designed (Table S1). Detailed methods are in Supplementary Materials 2.

**Table 1:** Primers used for PCR amplification and quantitative real-time PCR.

Name	Target gene	Primer sequence	Position/ amplified fragments	Application	Reference(s)
ppk1F	*ppk1*	GCN CGN TTY GAY GAR G	1378–1393^a^	PCR and quantification	This study
ppk1R	*ppk1*	TGY TCV AGR AAR CGN YC	1939–1955^a^	PCR	This study
ppk1qR	*ppk1*	TKG WAR TTN CCN GTN CC	1534–1550^a^	Quantification	This study
341F	16S rRNA	CCT ACG GGA GGC AGC AG	341–357^b^	Quantification	[33]
518R	16S rRNA	ATT ACC GCG GCT GCT GG	518–534^b^	Quantification	[33]
ppaF	*ppa*	GGCTCAATCCTCTGGCGTTA	197 bp	Quantification	This study
ppaR	*ppa*	CCATCATGGGCTGGTCCAAT		Quantification	This study
TUBA-F	tubulin α	TTCCTGGAGGAGACTTGGCTA	234 bp	Quantification	This study
TUBA-R	tubulin α	CAGCAGAGTCCACACCAACC		Quantification	This study

The primers used in qRT-PCR are ppk1F and ppk1qR. The schematic diagram of the *ppk1* gene and the primer design process are shown in Fig. S3.

^a^Numbering of the positions is based on the *ppk1* of *Mycobacterium tuberculosis* H37Rv.

^b^Numbering of the positions is based on the 16S rRNA gene of *Escherichia coli*.

### PCR amplification, sequencing, and data processing

The *ppk1* gene was amplified (ppk1F and ppk1R, Table 1) and sequenced (Pacbio Sequel II System). Amplification from January *H. simulans* samples failed due to extremely low gene abundance. We used the protein sequences translated from 620 full-length *ppk1* nucleotide sequences from NCBI as the reference. After being processed by FrameBot [34], 407 716 protein sequences were obtained. Operational protein units (OPUs) were clustered at 97% similarity. The number of *ppk1* gene sequences from each sample was rarefied to 21 620, which still yielded a Good’s coverage of more than 99.90%, confirming this depth was sufficient to recover most microbial diversity (Fig. S4). Detailed methods are in Supplementary Materials 2.

The eukaryotic *vtc4* gene of was amplified using three pairs of primers (Table S1). Clone library analyses of amplicons from primer pairs Vtc4-1F/Vtc4-1R and Vtc4-2F/Vtc4-2R were performed as previously described [15]. Illumina sequencing and data processing for amplicons from primer pair Vtc4-3F/Vtc4-3R followed established methods [32]. Sequencing was performed by Sangon Biotech (Shanghai) Co., Ltd., China.

### Sponge culture and thermal stress experiments

Due to the limited quantity of available specimens and their small individual size, *Tedania* sp. was excluded. In contrast, the more abundant *Spongia* sp. and *H. simulans* showed preliminary differences in PolyP accumulation and were selected for temperature experiments. Each healthy donor sponge was cut into three to six fragments, healed *in situ* for 30 days, and acclimated for 7 days in aquaria with artificial seawater. Heat stress experimental conditions simulated the present day and predicted for 2100 under Representative Concentration Pathway 8.5 scenarios (32°C) [35]: (i) 26°C (ambient temperature, used as a control), (ii) 30°C, and (iii) 32°C. For the control treatment (26°C), we used six fragments (3 fragments × 2 species). For the elevated temperature treatments (30°C and 32°C), we used 12 fragments (3 fragments × 2 species × 2 treatments). After reaching the experimental conditions, sponges were exposed to 30°C or 32°C for 1 week. The experiment was terminated for *H. simulans* after one day of exposure to 32°C due to extensive tissue necrosis in most samples. The total experiments duration was 27 days for *H. simulans* and 32 days for *Spongia* sp. Surviving sponges were rinsed, necrotic tissue removed, and healthy tissue was preserved in 75% (vol/vol) ethanol or RNAlater (4°C overnight, then −80°C) and stored at −80°C until use. Details in Supplementary Materials 2.

Temperature, salinity, dissolved oxygen (DO), pH, and phosphate concentration in the aquarium were monitored daily during the experiment. The measurement methods were performed as described previously [32].

### 16S rRNA gene sequencing and data processing

Genomic DNA was extracted from sponge tissues (*n* = 18, three replicates for each treatment per species) and sequenced by Biomarker Technologies Co., Ltd (Beijing, China). The V3-V4 region of the bacterial 16S rRNA genes was amplified with primer sets 338F (5′-ACTCCTACGGGAGGCAGCA-3′) and 806R (5′-GGACTACHVGGGTWTCTAAT-3′), and sequenced on a Novaseq System (Illumina, San Diego CA, USA). Our sequencing data showed that most of the bacterial diversity had recovered (Fig. S5). Bioinformatics analysis was performed using the BMK Cloud (Biomarker Techologies Co., Ltd). Methods for 16S rRNA gene amplicon data processing and analysis are in Supplementary Materials 2.

### RNA extraction, transcriptome sequencing, assembly, and annotation

Total RNA was extracted from 50 to 100 mg of sponge tissues using RNeasy Mini Kit (QIAGEN) following the manufacturer’s protocol. Contaminating DNA was removed using the PrimeScript RT reagent Kit with gDNA Eraser (Perfect Real Time, TaKaRa). RNA concentration and integrity were assessed using a Nanodrop 2000 spectrophotometer (Thermo Fisher Scientific, 02451, Waltham, MA, USA) and 1.5% agarose gel electrophoresis, respectively. Purified RNA was reverse transcribed immediately, and cDNA templates stored at −80°C.

For transcriptome sequencing, high-quality RNA from *Spongia* sp. and *H. simulans* tissues was sent to Biomarker Technologies Co., Ltd for sequencing. Libraries were prepared using high-quality RNA from three samples per treatment. RNA was reverse-transcribed using oligo (dT) primers to specifically enrich for host mRNA, thereby effectively filtering out most microbial sequences. Barcoded cDNA libraries were prepared and sequenced on the NovaSeq 6000 PE150 System (Illumina). A non-reference transcriptome sequencing analysis was performed on the sponge. Data assembly and annotation are provided in the Supplementary Materials 2.

### Quantitative real-time PCR analysis

Based on transcriptome data of the sponge host, qRT-PCR primers were designed targeting the inorganic pyrophosphatase (*ppa*) gene (Table 1). The tubulin α gene was selected as an endogenous reference for normalization in the sponge host [36], and specific primers were designed for this gene (Table 1). For quantification of *ppk1* expression in sponge-associated microorganisms, the 16S rRNA gene served as the reference across months (field experiment) and temperatures treatments (laboratory experiment) (Table 1). All reactions used pre-prepared cDNA templates. The qRT-PCR program and product verification procedures are detailed in Supplementary Materials 2.

## Results

### Seasonal dynamics of polyphosphate accumulation in sponges

PolyP accumulation showed distinct seasonal and interspecific variations. The highest PolyP concentrations were detected in summer (June), reaching 1.94 ± 0.28 mg/g in *Spongia* sp., 0.83 ± 0.30 mg/g in *Tedania* sp., and 0.18 ± 0.01 mg/g in *H. simulans* (Figure  1A). Lowest concentrations were recorded in winter (0.33 ± 0.03 mg/g in *Spongia* sp. in November, 0.17 ± 0.10 mg/g in *Tedania* sp., and 0.02 ± 0.01 mg/g in *H. simulans* in January). Furthermore, significant interspecific differences in PolyP concentrations were evident, with *Spongia* sp. accumulating two-fold more than *Tedania* sp. and 2–10-fold more than *H. simulans* during the same month (Figure  1A).

Seawater environmental factors fluctuated seasonally, with temperature showing opposite trends relative to DIP and DO concentrations (Fig. S6). Spearman correlation analysis showed that temperature was positively correlated with the PolyP accumulation of *Spongia* sp. and *Tedania* sp., but negatively correlated with DO (Figure  1B). *Spongia* sp. was also correlated with pH, DIP, and TP. In contrast, PolyP accumulation in *H. simulans* showed minimal correlation with all measured factors.

To verify PolyP presence, sponge tissue sections collected in June were visualized using DAPI-stained fluorescence microscopy. Excitation at 405-nm elicited characteristic green fluorescence emissions from DAPI-PolyP complexes, indicative of high PolyP accumulation. Consistent with the quantitative measurements, abundant green fluorescence particles were observed in tissues of *Spongia* sp. and *Tedania* sp., whereas only sparse particles appeared in *H. simulans* against a blue cellular background (Figure  1C).

### Seasonal variation in sponge *ppk1*-harboring microorganisms and *ppk1* gene expression

**Figure 2:**
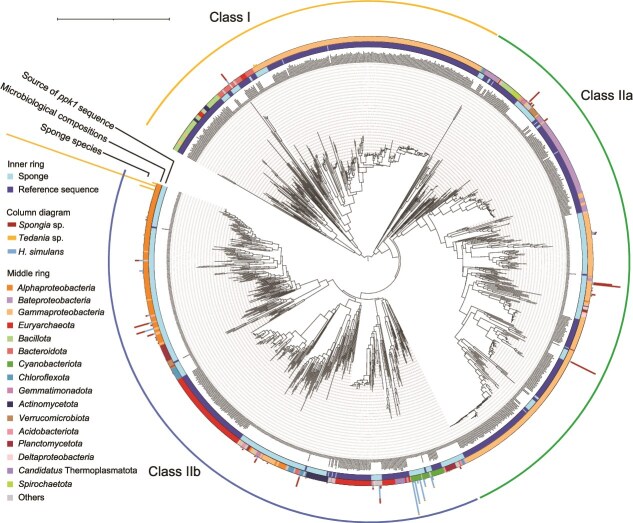
Maximum likelihood phylogenetic tree of *ppk1* amino-acid sequences from sponge samples and reference sequences from genomes. The inner ring indicates the source of the *ppk1* sequence. The middle ring indicates the species classification of each sequence. The histogram indicates the relative abundance of the OPUs in each sponge species. Branch length represents one nucleotide substitution per site.

A total of 620 *ppk1-*containing genomes were retrieved from the NCBI-Gene sequence database (June 2022). After removing highly similar sequences (> 99% amino acid similarity) within genera, 183 full-length sequences remained for primer design (Table 1), yielding a 578 bp fragment (Table 1, Fig. S3). Phylogenetic analysis of deduced protein sequences (620 sequences from the NCBI and 454 sequences from sponges in this study) revealed two distinct clades, which were defined as class I and II (Figure  2). Class I primarily comprised *Gammaproteobacteria, Bacillota, Bacteroidota*, and *Euryarchaeota*, with only 30 sequences originating from sponges. Class II contained sequences from diverse bacterial and archaeal phyla, including 424 sponge-derived bacterial sequences. The phylogenetic tree revealed that the primers used in this study amplified previously unidentified sponge-associated sequences clustering into *Alphaproteobacteria, Gammaproteobacteria, Chloroflexota*, and *Planctomycetota* but no archaeal *ppk1*-harboring groups were detected. Class II showed species-specific clustering, prompting us to subdivide it into class IIa (dominant in *Spongia* sp.) and IIb (dominant in *Tedania* sp. and *H. simulans*) for subsequent discussion. To evaluate the potential role of eukaryotic microbial symbionts in PolyP synthesis of the sponge holobiont, three primer pairs targeting the eukaryotic *vtc4* were designed to amplify the sequences (Table S2, Fig. S7). The failure to obtain amplification products suggests that phosphorus-accumulating capacity of sponges may be not primarily attributed to eukaryotic organisms, but rather to prokaryotes, particularly bacteria.

**Figure 3:**
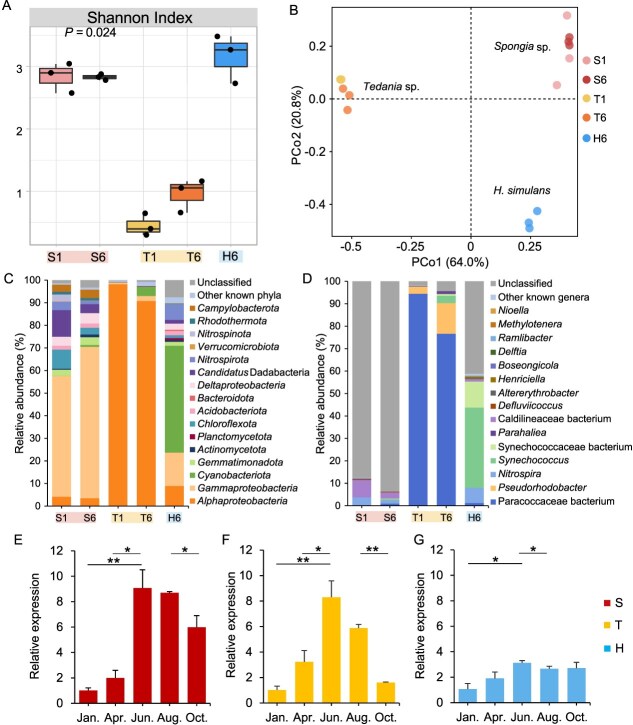
*ppk1*-harboring microbial community structure and gene expression across different months. (A) Shannon Index of *ppk1*-harboring microbial communities in different months. (B) Community structure. Principal coordinate analysis (PCoA) based on Bray–Curtis dissimilarity matrix. (C, D) Relative abundance of microbial phyla (C) and genera (D). (E-G) *ppk1* gene expression in different months. S = *Spongia* sp., T = *Tedania* sp., H = *H. simulans*. Arabic numerals represent months. Significance levels: ^*^: *P* < .05, ^**^: *P* < .01.

The *ppk1*-harboring microbial assemblages in samples collected in June (peak PolyP accumulation) and January (minimal PolyP accumulation) were further analyzed (Figure  3). Shannon Index was highest for *H. simulans* (3.2), followed by *Spongia* sp. (2.8) and *Tedania* sp. (0.7) (Figure  3A). PCoA analysis revealed compositional differences among sponge species (Figure  3B). In *Spongia* sp., the dominant PolyP accumulation taxa were *Gammaproteobacteria* (average relative abundance, 60.2%), *Candidatus* Dadabacteria (7.9%), and *Chloroflexota* (5.7%). *Alphaproteobacteria* (94.5%) was the dominant taxon in *Tedania* sp. *Cyanobacteriota* (47.3%), *Nitrospirota* (6.8%), and *Gammaproteobacteria* (14.7%) accounted for most of the *ppk1*-harboring microbial assemblages in *H. simulans*. At the genus level, most taxa in *Spongia* sp. remained unclassified, with minor contributions from Caldilineaceae bacterium (5.2%) and *Nitrospira* sp. (2.6%). Paracoccaceae bacterium (85.6%) was the predominant taxon in *Tedania* sp., whereas *Synechococcus* sp. (35.7%), Synechococcaceae bacterium (11.6%), and *Nitrospira* sp. (6.8%) were the dominant group of *H. simulans*. Compared with sponge-associated bacterial community (based on previously published 16S rRNA sequencing data [32]), the *ppk1*-harboring microbial assemblages were confirmed to be dominant constituents of the sponge microbiota (Fig. S8).

The *ppk1*-harboring microbial composition remained largely stable across seasons, with minor phylum- and genus-level fluctuations observed, except for *H. simulans* in January where *ppk1*-PCR amplification failed (Figure  3C and D, Fig. S9). In contrast, *ppk1* gene expression varied seasonally, peaking in June and reaching minima in January across species (Figure  3E–G). Specifically, compared to January, the expression of the *ppk1* gene in June was nine-fold higher in *Spongia* sp., 8.1-fold higher in *Tedania* sp., and three-fold higher in *H. simulans*. This *ppk1* gene transcription trend aligned with the annual PolyP accumulation dynamics (Figure  1A). Furthermore, *Spongia* sp. consistently showed approximately two and three-fold higher *ppk1* expression than *Tedania* sp. and *H. simulans*, respectively, consistent with its higher PolyP accumulation capacity.

### Effects of simulated warming on sponge bacterial communities

**Figure 4:**
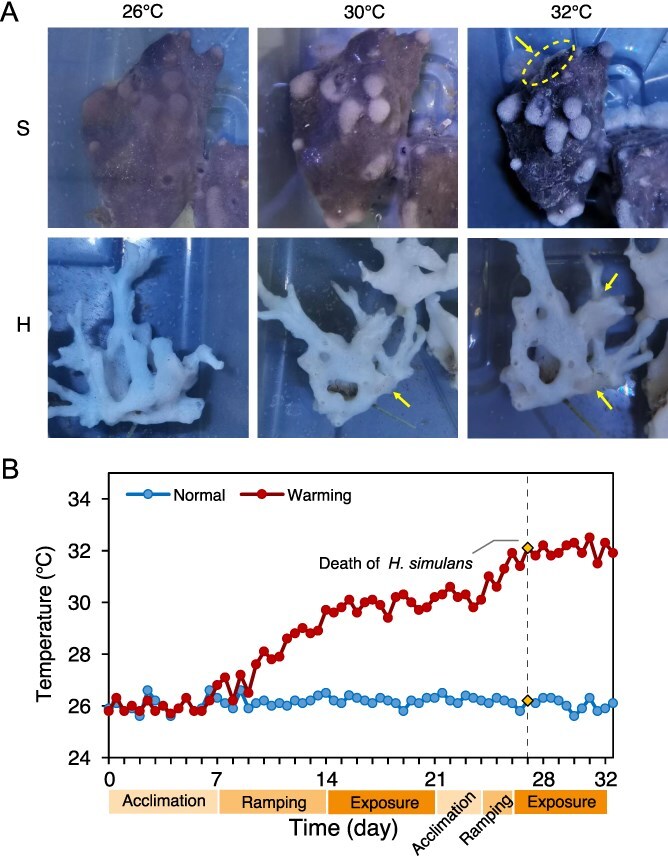
Pictures of sponges (A) under heat stress, and physico-chemical parameters throughout the duration of experiment (B). S = *Spongia* sp., H = *H. simulans*. Arrows indicate slight tissue discoloration and exposed skeleton of *Spongia* sp. In *H. simulans*, tissue exhibits a bleached appearance​ as temperature increases, and the arrow indicates the area of necrotic tissue.

*Spongia* sp., showed greater thermal adaptation, with no tissue necrosis or mortality throughout the experimental period. Compared with the control group (26°C), prolonged exposure to 32°C induced minor physiological responses in *Spongia* sp., including slight mucus secretion and localized skeletal exposure (Figure  4A). At the control temperature, *H. simulans* exhibited a healthy pale blue-gray coloration. However, raising the temperature to 30°C caused tissue bleaching and localized necrosis, and 32°C caused extensive necrosis and near-total pigment loss within 2 days (Figure  4).

**Figure 5:**
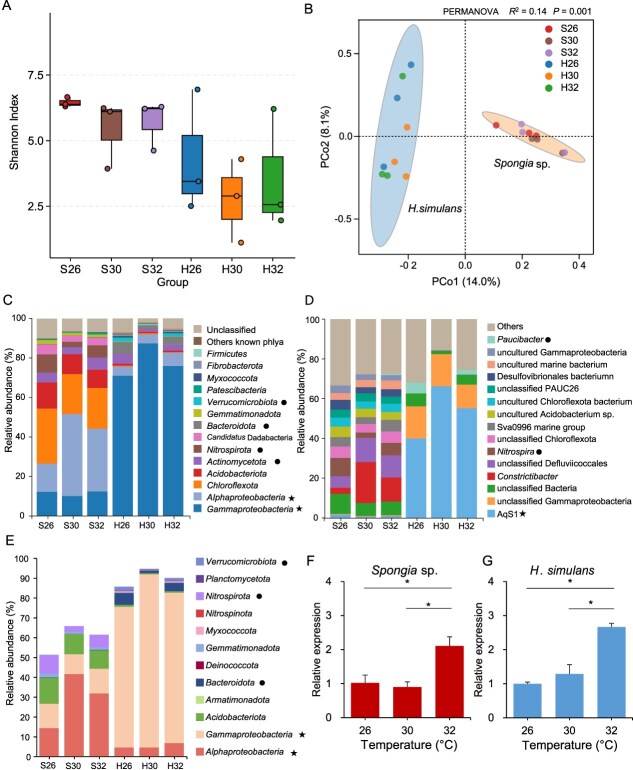
Sponge-associated bacterial community structure under heat stress. (A) Shannon Index under different temperature stresses. (B) Bacterial community structure. Principal coordinate analysis (PCoA) based on Bray–Curtis dissimilarity matrix. (C, D) Relative abundance of bacterial phyla (C) and genera (D). (E) Relative abundance of *ppk1*-harboring bacterial phyla. ★ and ● indicate taxa whose relative abundance increased then decreased, or decreased then increased, respectively, with increasing temperature. S = *Spongia* sp., H = *H. simulans*. Numbers indicate the exposure temperature. (F, G) Expression of the *ppk1* gene in sponge-associated microorganisms at different temperatures in *Spongia* sp. (F) and *H. simulans* (G). Significance levels: ^*^: *P* < .05.

Bacterial alpha diversity (Shannon Index) in both sponge species showed no significant change (*t* test, *P* > .05) with increasing temperature, despite a slight downward trend, and was consistently higher in *Spongia* sp. than *H. simulans* (Figure  5A). PERMANOVA analysis confirmed a significant community separation between the two species (*R*^2^ = 0.14, *P* = .001) (Figure  5B). However, temperature elevation did not significantly alter the overall bacterial community structure within each species (*P* = .252 for *Spongia* sp., *P* = .467 for *H. simulans*) (Figure  5C and D, Fig. S10). The bacterial communities remained distinct host-specific: *Spongia* sp. was characterized by *Alphaproteobacteria* (30.1%), *Chloroflexota* (22.8%), *Gammaproteobacteria* (11.5%), and *Acidobacteriota* (10.8%), whereas *H. simulans* was dominated by *Gammaproteobacteria* (78.2%). Although the bacterial composition in both sponges did not change significantly (Fig. S11), several taxa exhibited abundance fluctuations, with a notable shift observed at 30°C. Host-specific bacteria, such as *Constrictibacter* sp. (*Alphaproteobacteria*) within *Spongia* sp. (relative abundance: 26°C = 3.1%, 30°C = 20.5%, 32°C = 12.1%) and AqS1 (*Gammaproteobacteria*) in *H. simulans* (26°C = 39.9%, 30°C = 66.2%, 32°C = 55.2%), initially increased in abundance before declining. Conversely, *Nitrospirota* and *Nitrospira* sp. within *Spongia* sp., as well as *Actinomycetota, Bacteroidota*, and *Paucibacter* sp. in *H. simulans*, displayed an opposite trend.

By cross-referencing the taxonomic identities of the *ppk1*-harboring populations (Figure  3) with the relative abundance profiles from the 16S rRNA dataset of sponge-associated bacterial community, we tracked the dynamics of these specific *ppk1*-associated groups within the bacterial community under warming conditions. Extracted *ppk1*-harboring microbial assemblages exhibited similar dynamic compositional shifts across temperature (Figure  5E, Fig. S12). Although the *ppk1*-harboring microbial assemblages at phylum and genus levels, were not significantly perturbed by temperature (*t* test, *P* > .05), special bacterial taxa changed. The relative abundance of *Alphaproteobacteria* first increased and then decreased (26°C = 14.3%, 30°C = 41.5%, 32°C = 31.8%) in *Spongia* sp*.* In contrast, *Bacteroidota* in *H. simulans* exhibited the opposite trend (26°C = 6.0%, 30°C = 1.3%, 32°C = 4.2%). At the genus level, *Vibrio* sp. increased from 26°C (0.0%) to 30°C (0.8%) and then decreased at 32°C (0.05%), whereas *Endozoicomonas* sp. showed a gradual increase in relative abundance with rising temperature in *H. simulans* (26°C = 0.04%, 30°C = 0.3%, 32°C = 0.5%) (Fig. S12). The *ppk1* gene expression in sponge-associated microorganisms increased with rising temperature (Figure  5F and G). In both sponge species, the *ppk1* expression level at 32°C was 2–3 times higher than at 26°C.

### Transcriptomic responses of sponge hosts to simulated warming

**Figure 6:**
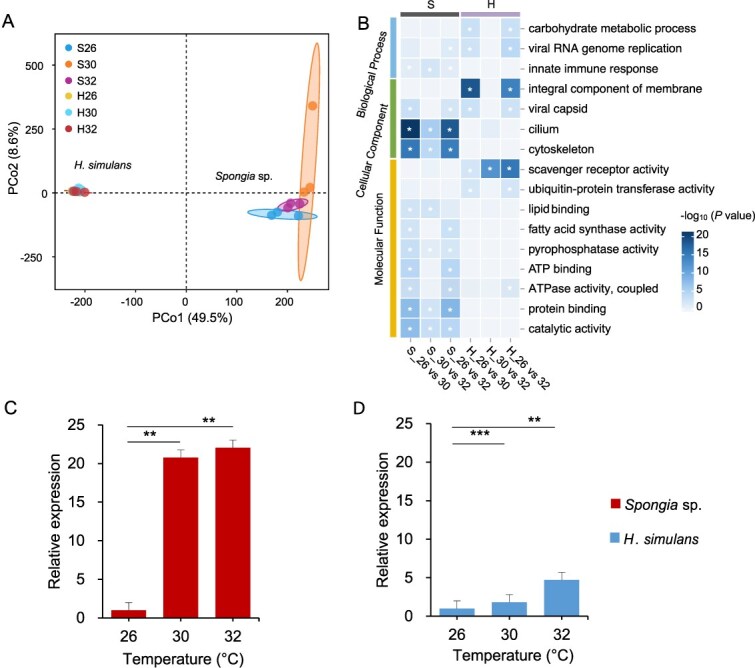
Transcriptome profile of the sponge host under in response to thermal stress. (A) PCoA plot based on the KEGG Orthology terms (KOs) of sponge host. (B) Gene Ontology (GO) enrichment analysis for transcripts in *Spongia* sp. and *H. simulans* under different temperature regimes. (C, D) *ppa* gene expression in sponge hosts at different temperatures. S = *Spongia* sp., H = *H. simulans*. Numbers indicate the exposure temperature. Significance levels: ^*^: *P* < .05, ^**^: *P* < .01, ^***^: *P* < .001.

Sponge transcriptome assembly yielded 99 863 transcripts (N50 = 2002) with quality comparable to other poriferan transcriptomes [26]. Despite gene expression clustered by the same sponge species across temperatures, substantial differentially expressed genes (DEGs) were identified (Figure  6A, Fig. S13). *Spongia* sp. exhibited significantly more DEGs than *H. simulans* in response to warming, especially at 30°C. In sharp contrast, 1028 DEGs were detected in *Spongia* sp. between 30°C and 32°C, compared to only 143 DEGs in *H. simulans*.

Gene Ontology (GO) enrichment revealed species-specific functional responses to warming. DEGs in *Spongia* sp. were enriched in cilium, cytoskeleton, protein binding, and catalytic activity, reflecting active cell growth and metabolism (Figure  6B). Conversely, in *H. simulans*, DEGs were enriched for carbohydrate metabolic process, integral component of membrane, scavenger receptor activity, and sulfuric ester hydrolase activity (Figure  6B). Elevated viral RNA replication signatures suggested progressive inflammation. Based on DGEs *Spongia* sp. exhibited stronger innate immune response under heat stress, and exhibited significantly higher gene expression associated with lipid metabolism (e.g. lipid binding and fatty acid synthase activity) as well as oxidative phosphorylation (pyrophosphatase activity and ATP binding). With increasing temperature, *ppa* gene expression increased, with levels significantly higher in *Spongia* sp. than *H. simulans* (Figure  6C and D). Both sponges significantly enhanced ATPase activity at 32°C, with *Spongia* sp. showing particularly enhanced metabolic activity (e.g. protein binding and catalytic activity).

KEGG analysis highlighted divergent stress response strategies. *Spongia* sp. prioritized extracellular matrix (ECM)–receptor interactions and cysteine/methionine metabolism, with apoptotic signals intensifying at 32°C. In contrast *H. simulans* DEGs clustered in pathways related to ubiquitin-mediated proteolysis, lysosomal function, glycosaminoglycan degradation, and TGF-β signaling at both 30°C and 32°C (Fig. S14).

## Discussion

Beyond its geological sequestration role [16], microbially synthesized PolyP enhances cellular resistance to environmental stress [17, 18]. Sponge symbionts produce and store this polymer [14], potentially vital for holobiont survival under future ocean conditions. By integrating field surveys with controlled experiments, this study demonstrated that elevated temperature influences sponge PolyP accumulation, with marked interspecific differences. We found that changes in holobiont PolyP levels were associated primarily with microbial *ppk1* gene expression, rather than shifts in microbial composition, suggesting a potential mechanism by which the sponge holobionts respond to ocean warming.

### Host-specific *ppk*1-harboring consortia mediate polyphosphate accumulation

Sponges exhibit exceptional environmental adaptability, reflected in their evolutionary persistence and wide distribution [37, 38]. Our study revealed that this resilience was linked to their symbiotic microorganisms, as evidenced by sponge species-specific differences in PolyP accumulation. We identified that host-specific microbial assemblages harboring the *ppk1* gene were key drivers of PolyP accumulation in sponges. Our new PCR primers targeting *ppk1* revealed more sponge-specific sequences, expanding the known genetic diversity of this critical enzyme in marine symbioses. These *ppk1*-harboring communities are host-specific across different sponge species, mirroring the overall pattern of microbiome assembly [32]. Although *Cyanobacteriota* have been identified as primary PolyP contributors in some reef sponges [14, 15, 23], our findings challenge their presumed universal role. *H. simulans*, despite hosting abundant *Cyanobacteriota*, exhibits minimal PolyP accumulation corroborated by the low *ppk1* gene expression. Conversely, *Tedania* sp. and *Spongia* sp., demonstrated high PolyP accumulation and were dominated by *ppk1*-harboring *Alphaproteobacteria* and *Gammaproteobacteria*, respectively. This contrast indicates that the specific *ppk1*-harboring microbiota determines interspecific variation in PolyP storage capacity. Furthermore, we observed that although PolyP levels fluctuated seasonally and with elevated temperature stress (32°C), the *ppk1*-harboring assemblages remained stable. This stability implies that differential *ppk1* gene expression underpins PolyP fluctuations. These findings highlight the capacity of sponge-associated microorganisms for physiological adaptations that may confer environmental stress adaptation on the holobiont.

Although elevated temperatures have been reported to perturb the sponge microbiome [29], our results indicate that bacterial communities exhibited structural stability with some degree of plasticity, as certain taxa showed abundance trends at 32°C toward the control group (26°C). This maintenance of core bacterial community structure aligns with observations in other thermally stable sponge species [39], such as the Great Barrier Reef sponge *Rhopaloeides odorabile* [40], and Mediterranean sponge *Ircinia* sp. [41, 42]. Although not statistically significant, the increase in health-associated Alphaproteobacterium [43] in *Spongia* sp. under warming correlated with the host’s higher thermal adaptability. Similarly, the proliferation of the commensal bacterium AqS1 in stressed *H. simulans* may reflect a functional role in supporting host fitness, possibly by enhancing nutrient utilization [44]. Our data suggest that bacterial communities possibly continue to play a positive role even during host physiological decline. The functional activity of bacterial communities may change markedly through mechanisms such as transcriptional regulation or fine-tuning of metabolic pathways that warrant further investigation using metatranscriptomic and metabolomic approaches in future.

### Sponge host exhibit divergent physiological responses to future climate

Our findings revealed distinct physiological trajectories for sponges under simulated future warming, potentially reflecting fundamental differences in their holobiont configuration. *Spongia* sp. exhibited higher thermal tolerance, correlating with significant upregulation of genes involved in ECM–receptor interactions, innate immunity, lipids/fatty acids synthesis, and energy metabolism (increased *ppa* gene expression, ATP binding, and ATPase activity) [45]. These coordinated responses for enhanced cell communication, structural integrity, protective metabolite production, and energy mobilization, reflect a adaptative pattern previously associated with thermal resilience in sponges [26, 46]. Conversely, the LMA sponge *H. simulans* suffered extensive tissue necrosis under heat stress, concurrent with host upregulation of pathways including TGF-β signaling pathway [47], lysosomal activity, proteolysis, immune function receptor activity, indicative of cell cycle arrest, apoptosis, and detrimental immune activation. Furthermore, the observed upregulation of RNA virus replication processes likely exacerbates this response, collectively suggesting a possible contribution to cellular damage.

These divergent molecular responses may reflect underlying differences in microbial symbiosis and host physiology between the two species [32, 48]. The HMA sponge *Spongia* sp., a sponge characterized by slower filtration rates and denser mesohyl matrix, may possess greater intrinsic protective capabilities [32, 48, 49]. The host physiological traits such as filtration capacity and tissue density along with symbiotic bacterial community composition, possibly mediate the holobiont’s response to thermal stress. In contrast, the LMA sponge *H. simulans*, with rapid seawater pumping and lower tissue density, experiences greater environmental exposure [32, 48, 49]. This may elevate pathogen encounter rates and diminish physical buffering capacity, potentially accounting for the observed viral activity and heightened yet maladaptive immune response. The capacity of *Spongia* sp. for coordinated gene expression of structure maintenance, metabolism adjustment, and innate defense mechanisms likely underpins its thermal adaptation [50]. These findings suggest that integrative molecular plasticity, shaped by host–microbe partnerships and species-specific physiological traits, influences holobiont viability under future ocean conditions.

### Microbially derived polyphosphate potentially supports sponge holobionts in response to climate change

PolyP synthesis represents a conserved microbial response to environmental stress and serves as an early indicator in stress regulation [18]. PolyP may serve as a functional indicator of holobiont environmental response, as its fluctuation aligns with stress. This pattern parallels observations in Lake Ontario phytoplankton, where PolyP accumulation peaks during summer [25], underscoring PolyP’s universal role in stress adaptation. Our findings revealed substantial energy consumption in sponges during heat stress, evidenced by strongly elevated expression of host ATP-related enzymes. In *Spongia* sp., the significant upregulation of *ppa* and ATP binding activity suggests an increased demand for ATP synthesis under thermal stress. The absence of *ppk1* (prokaryotes PolyP synthesis) nor *vtc4* (eukaryotic PolyP synthesis) genes in host transcriptome of both studied sponges as well as in other sponge genomes such as *Amphimedon queenslandica* [51], implies that PolyP accumulated by symbiotic bacteria (by *ppk1*) may provide a phosphate source for host oxidative phosphorylation, thereby supporting host ATP synthesis (*ppa*, Fig. S15). Although microbial nutrient conversion for host is well-established in other symbioses (e.g. gut bacteria and mycorrhizae) [52, 53], the direct microbial storage of nutrients such as PolyP for host utilization appears to be a distinctive feature of sponge-microbe partnerships [22].

Beyond energy storage, PolyP regulates the transcriptional regulation of cellular processes including growth, proliferation, and apoptosis [17]. Under thermal stress causing proteins denaturation [54], microbial PolyP can bind to heat shock proteins, preventing protein inactivation, and thereby enhancing microbial thermotolerance [55]. Furthermore, PolyP interacts with proteins, nucleic acids, and membranes, broadly modulating enzyme activity, stress responses, and cellular homeostasis [17–20]. Given these functions, the substantial PolyP synthesized by sponge-associated microorganisms may thus directly or indirectly affect host physiology. In *Spongia* sp., upregulation of genes associated with cytoskeleton, cilium, protein binding, and catalytic activity in the host, alongside abundant PolyP accumulation, suggested possible coordinated regulation of holobiont integrity. The contrasting responses of *Spongia* sp. and *H. simulans* to thermal stress revealed a potential link between PolyP dynamics and holobiont resilience. *Spongia* sp. maintained high and steadily increasing PolyP accumulation, effectively meeting both energy (via ATP synthesis) and cellular protective requirements. Conversely, *H. simulans* severe cellular damage with declining PolyP reserves despite upregulated microbial *ppk1* expression at 32°C. This divergence suggests that under intense thermal stress, the rate of PolyP consumption in *H. simulans* may exceed its replenishment capacity. The stability of *ppk1*-harboring microbial assemblages amid dynamic gene expression highlights that functional plasticity, rather than structural reorganization, mediates short-term PolyP dynamics. PolyP accumulation may buffer against heat stress, contributing to holobiont survival.

**Figure 7:**
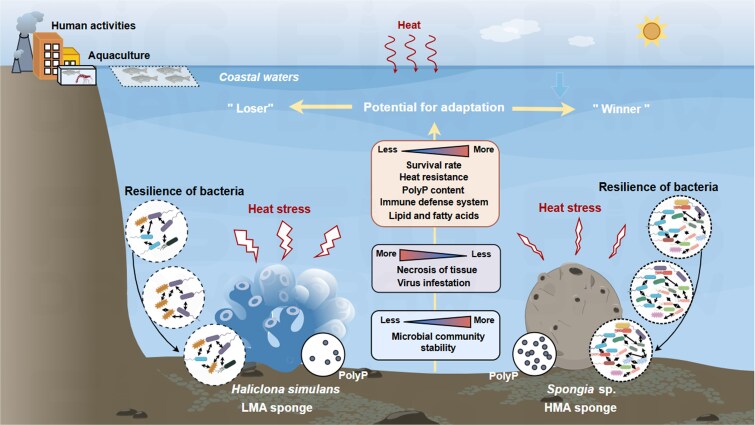
There are differences in the adaptation potential of *Spongia* sp. and *H. simulans* in future coastal environments. The properties of holobionts determine the viability of sponges in future environments. Figure created using Figdraw.

Although sponges are considered resilient to future climate conditions [7], their responses to warming vary considerably among species. Our results suggest that microbially mediated PolyP accumulation may represent an important mechanism contributing to the adaptability of sponge holobionts to ocean warming. HMA sponges such as *Spongia* sp. which harbor more abundant and diverse microbiomes than LMA sponges *such as H. simulans*, are associated with complex holobiont functions including nutrient acquisition and chemical defense [56, 57]. The contrasting responses observed between *Spongia* sp. and *H. simulans* raise the possibility that HMA and LMA sponges may differ in their capacity to maintain microbially derived PolyP reserves under thermal stress (Figure  7). Whether robust PolyP accumulation is a consistent trait of HMA symbioses and whether it confers a competitive advantage under ocean warming, are key questions for future study.

## Conclusion

This study identified microbially derived PolyP as a potential mediator of sponge holobiont resilience to ocean warming. Our findings indicate that responses of sponges to ocean warming are linked to host-specific microbial consortia, which regulate PolyP accumulation primarily through *ppk1* gene expression. The divergent responses of *Spongia* sp. and *H. simulans* suggest that species-specific holobiont characteristics, particularly the ability to sustain PolyP homeostasis under stress, may shape survival trajectories. Although sponges are considered relatively tolerant to climate change, our results highlight that their persistence could associated with specific functional microbiome partnerships and host species identity. Therefore, we propose that microbe-mediated PolyP dynamics can serve as a valuable indicator for predicting sponge resilience under ongoing climate change. Although *H. simulans* exhibited necrosis and mortality under high temperatures, its bacterial community showed only limited changes warranting further investigation. Furthermore, expanding such analyses to a broader range of sponge species will help elucidate the general mechanisms underlying holobiont adaptation to environmental stress, thereby improving prediction of future community structure and distribution of coral reefs.

## Supplementary Material

Supplementary_material_wrag128

## Data Availability

Raw sequences of *ppk1*-harboring microbial assemblages, sponge microbiome, and host transcriptome were deposited in the Sequence Read Archive (SRA) operated by the NCBI under BioProject accession number: PRJNA1205819, PRJNA1188811 and PRJNA1206773, respectively.
