# Supplementary material for: Microbiome-mediated polyphosphate accumulation enhances the resilience of sponge holobionts to future climate scenarios
Source: ISME J. 2026 May 29;20(1):wrag128. doi: 10.1093/ismejo/wrag128 (PMC13431277; doi:10.1093/ismejo/wrag128)
Supplement: Supplementary_material_wrag128 [file supplementary_material_wrag128.zip › Supplementary materials 2_v.docx]

**Microbiome-mediated polyphosphate accumulation enhances the resilience of sponge holobionts to future climate scenarios**

**Supplementary materials and methods**

**Extraction and measurement of total PolyP from sponge tissue**

PolyP was extracted and measured from the sponge tissue according to a previously published method [1]. Sponge tissues from three replicate individuals of each species were lyophilized. Approximately 2-5 mg of the lyophilized tissue was then finely ground, suspended in 20 mM Tris buffer (pH 7.0) and vortexed. The suspension was subsequently sonicated for 1 min, immersed in boiling water for 5min, on ice for 5min, and sonicated again for 1min. The samples were treated with 5 units/µL DNase I (TaKaRa) and 10mg/ml RNase A (TaKaRa) at 37°C for 10 min to remove DNA and RNA. Proteinase K (20 mg/mL, TaKaRa) was added and incubated at 37°C for 30 min, centrifuged at 12000 rpm for 1 min, and the supernatant was collected. Four additional consecutive extractions of sponge tissue were performed following the same protocol. Supernatants collected from the previous four extractions were incubated with DAPI (the final concentration was 10 µm), which were used for PolyP fluorescence assays. In the last extraction, the fluorescence produced by the interaction of DAPI with other substances was background fluorescence. Fluorescence measurement was performed on a Tecan fluorometer (Infinite M200 PRO, Switzerland). The excitation wavelength was set to 415 nm and emission spectra were recorded from 450 to 600 nm in 2 nm increments. Upon UV excitation, the binding of PolyP to DAPI shifts the peak emission wavelength of DAPI from 475 to 550 nm, and the fluorescence intensity at this shift wavelength is proportional to the PolyP concentration [1]. PolyP concentration was quantified against a standard curve generated using a PolyP standard (S4379, Sigma–Aldrich) and normalized to the sponge tissue mass. The PolyP standard curve was shown in Fig. S2.

**Visualization of** **PolyP granules by confocal microscopy**

Sponge tissues samples (about 0.2 cm^3^) were washed in a a graded ethanol series embedded in paraffin, and then sectioned. Section staining were performed as described previously [2]. The sections were sealed using DAPI quencher resistant resin. They were visualized under a Zeiss LSM780NLO (Carl Zeiss AG, 73447, Oberkochen, Germany) inverted confocal microscope equipped with a 40 × Axio Observer Z1 automatic inverted fluorescence/lens (1.3N.A.). Sample sections were visualized by excitation from a 405 nm laser source, and the emission signals were separated by an NFT515 filter into two channels to collect DAPI-nucleotide and DAPI-PolyP signals, respectively. The emission wavelengths collected in channel 1 were 420–515 nm, representing the nucleotid–DAPI signal. Channel 2 was used to collect emission wavelengths above 530 nm representing the PolyP–DAPI signal. The merged images of the two channels are shown in Fig. 1.

**Design of primers for *ppk1* gene and validation**

Full-length *ppk1* nucleotide sequences from prokaryotic genomes were obtained from the National Center for Biotechnology Information (NCBI) database. These sequences were translated, and aligned at the amino acid level using the BioEdit (https://github.com/thalljiscience/thalljiscience.github.io). Based on the alignment of full-length amino acids, the corresponding nucleotide alignment was used for primer design. WebLogo 3 (https://weblogo.threeplusone.com/) was used to simplify alignment results. Several sets of degenerate primers for several sets of *ppk1* sequences were designed and tested against sponge species known to have *ppk1* from previous study [2]. PCR reaction conditions were optimized for all sponge-associated microorganisms containing the *ppk1*. The genomic DNA was extracted directly from the hosts following the previously described method [2], and DNA was stored at −80°C until use. All reactions were performed using 2×Hieff Canace PCR Master Mix [Yeasen Biotechnology (Shanghai) Co., Ltd, China]. Thermal cycling conditions were an initial 5 min denaturing step at 98°C, followed by 35 cycles of 98°C for 10 s, a gradient annealing of 48 to 65°C for 30 s, 72°C extension for 30 s and a final extension at 72°C for 10 min. The experimental results indicated that the primers ppk1F and ppk1R were the best candidate primers (Table 1), and the optimal annealing temperature was 56°C. The same method was used for primer design for quantitative real-time PCR (qRT-PCR), primers ppk1F and ppk1qR (Table 1) yielded the strongest and most specific amplification products and were selected as the optimal primers. Applying the same primer design strategy, multiple primer pairs targeting the eukaryotic *vtc4* gene (involved in PolyP synthesis such as in organisms fungi and algae) were also designed. Three candidate *vtc4* primer pairs were validated by conventional PCR (Table S1).

***ppk1* gene sequencing and data processing**

The primers ppk1F and ppk1R were used for amplification. Triplicate PCR products for each sample were pooled and verified by 1.8% (w/v) agarose gel electrophoresis to confirm specificity. PCR products were purified and sequenced on the Pacbio Sequel II System (Pacific Biosciences, CA, USA) by Majorbio Bio-Pharm Technology Co., Ltd. (Shanghai, China). Raw sequencing data were processed using the SMRT Link (v8.0) to obtain demultiplexed circular consensus sequence (CCS) reads with a minimum of three full passes and 99% sequence accuracy. CCS reads were barcode-identified and length-filtered. FrameBot was used to check and correct the frameshift errors with CCS reads [3]. The protein sequences were clustered into operational protein units (OPUs) using UPARSE 7.1 [4] with 97% sequence similarity level. The taxonomy of each OPU representative sequence was subjected to a BLASTP search against the National Center for Biotechnology Information (NCBI) non-redundant protein sequences (nr) database (<https://ftp.ncbi.nlm.nih.gov/blast/db/FASTA/>).

**Sponge culture and stress response experiments**

Each healthy donor sponge (*Spongia* sp. and *H. simulans*) was cut into 9 fragments using a sterile razor. Among them, the block-shaped *Spongia* sp. was cut into 50 cm^3^ fragments and the dendritic *H. simulans* was cut into fragments about 10 cm long and 5 cm wide. All sponge fragments were placed in situ and allowed to heal for 30 days. After the healing period, the fragments were taken back to the laboratory and acclimated for 7 days in aquaria containing aerated artificial seawater maintained at pH 8.0 and 26°C.

Stress response experiments were performed in 30L independently aerated aquaria. Temperature was regulated using a 100W submersible heater, and illumination followed a 12:12 light:dark photocycle. Treatment conditions included (i) 26°C, (ii) 30°C, and (iii) 32°C. Temperature was changed gradually (+0.5°C/day) until the desired conditions were reached. Sponges were exposed to 30°C or 32°C for a week. The experiment was terminated for *H. simulans* after one day of exposure to 32°C due to extensive tissue necrosis in most samples. The total experiments duration was 27 days for *H. simulans* and 32 days for *Spongia* sp. Surviving sponges were rinsed with filtered artificial seawater, necrotic tissue was removed, and a portion of healthy tissue was perserved in 75% (vol/vol) ethanol at –80°C. Another part was immersed in RNAlater overnight at 4°C and subsequently stored at –80°C until use.

**16S rRNA gene data processing**

Data processing and analysis of 16S rRNA was performed using QIIME2 (version 2020.6) [5]. Chimeric sequences and singletons were removed from raw data, and amplicon errors were corrected using the DADA2 package [6], and amplicon sequence variants (ASVs) were identified and tabulated. The ASVs with relative abundace <0.005% were filtered. After archaeal sequences were removed and the data were normalized, we obtained 4,971,760 high-quality bacterial sequences clustered into 8,431 ASVs. Taxonomy annotation of the ASVs was performed based on the Naive Bayes classifier using the SILVA ribosomal reference database release 132 (<http://www.arb-silva.de/>).

**Transcriptome assembly, and annotation**

Reads with low-quality scores or contained adapter sequences were removed from the raw data. We obtained a total of 124.55 Gb Clean Data, and the percentage of Q30 base in each sample was not less than 93.34%. De novo transcriptome assembly was carried out using Trinity (v3.1b2) [7]. In the subsequent analyses, publicly available sponge genomes and invertebrate databases were consulted. Additionally, a random selection of 2,000 reads was aligned against the NT database, with the results indicating no detectable microbial sequence information. Unigenes were annotated by alignment against the NR [8], Swiss-Prot [9], COG [10], KOG [11], eggNOG4.5 [12] and KEGG [13] databases using DIAMOND (v2.0.4) [14]. The amino acid sequence of the unigenes,were predicted and then searched against the Pfam database [15] using the HMMER (v3.1b2) [16] for domain annotation.

**Quantitative real-time PCR (qRT-PCR) analysis**

The qRT-PCR program for all genes included an initial denaturation step of 35 s at 95°C, followed by 45 cycles of denaturation for 5 s at 95°C, annealing for 50 s at 55°C, and extension for 50 s at 72°C. Fluorescence signals were collected at 72°C, and negative controls lacking template DNA were subjected to the same qRT-PCR procedures to monitor possible contamination. Melting curve analysis and 2% agarose gel electrophoresis were used to confirm the specificity of qRT-PCR products. Melting curves were acquired by heating from 85 to 95°C, with a read every 1°C and holding for 1 s between reads. All qRT-PCR assays were carried out in triplicate with an ABI Prism 7500 sequence detection system (Applied Biosystems), following SYBR green qRT-PCR protocols. The resultant qRT-PCR data were analyzed with the second derivative maximum method using the QuantStudio Real-Time PCR Software (version 1.3; Applied Biosystems).

**Bioinformatic and statistical analyses**

**Statistical analyses**

The sequences were further processed with a bioinformatic pipeline tool, BMKCloud (www.biocloud.net) online platform. The similarity among the microbial communities in different samples was determined by principal coordinate analysis (PCoA) based on Bray-curtis dissimilarity. Permutational multivariate analysis of variance (PERMANOVA) test was used to assess the percentage of variation explained by the treatment along with its statistical significance [17]. The significance of differences between different samples was tested using the Wilcoxon test. Correlation analysis was based on the Spearman method. P <0.05 was considered statistical significance.

**Phylogenomic tree**

To reveal the *ppk1* sequence diversity in symbionts, all genomes encoding *ppk1* (620) were downloaded from the NCBI Gene database. Multiple sequence alignments were generated for the PPK1 protein sequences from these symbiotic sources, along with reference *ppk1* sequences from selected genomes, using the MAFFT [18]. Then, an unrooted maximum-likelihood phylogenetic tree was constructed from the alignment using FastTree2 [19]. The generated tree in newick format was visualized using iTOL v6 (<https://itol.embl.de/>).

**Reference**

1. Zhang F, Blasiak LC, Karolin JO. et al. Phosphorus sequestration in the form of polyphosphate by microbial symbionts in marine sponges. *Proc Natl Acad Sci USA* 2015; 112**:**4381-4386.

2. Ou H, Li M, Wu S. et al. Characteristic microbiomes correlate with polyphosphate accumulation of marine sponges in south china sea areas. *Microorganisms* 2020; 8**:**63.

3. Wang Q, Quensen John F, Fish Jordan A. et al. Ecological Patterns of *nifH* genes in four terrestrial climatic zones explored with targeted metagenomics using FrameBot, a new informatics tool. *mBio* 2013; 4**:**e00592-00513.

4. Edgar, R.C., UPARSE: highly accurate OTU sequences from microbial amplicon reads*.* *Nat Methods* 2013;10:996-8.

5. Bolyen, E., J.R. Rideout, M.R. Dillon, N.A. Bokulich, C.C. Abnet, G.A. Al-Ghalith, et al., Reproducible, interactive, scalable and extensible microbiome data science using QIIME 2*.* *Nat Biotechnol* 2019; 37:852-857.

6. Callahan BJ, McMurdie PJ, Rosen MJ. et al. DADA2: High-resolution sample inference from Illumina amplicon data. *Nat Methods* 2016;13**:**581-583.

7. Haas BJ, Papanicolaou A, Yassour M. et al. De novo transcript sequence reconstruction from RNA-seq using the Trinity platform for reference generation and analysis. *Nat Protoc* 2013;8**:**1494-1512.

8. Deng YY, Li JQ, Wu SF. et al. Integrated nr database in protein annotation system and its localization. *Comput Eng* 2006;32**:**71-72.

9. UniProt Consortium T. UniProt: the universal protein knowledgebase. *Nucleic Acids Res* 2018;46**:**2699.

10. Tatusov RL, Galperin MY, Natale DA. et al. The COG database: a tool for genome-scale analysis of protein functions and evolution. *Nucleic Acids Res* 2000;28**:**33-36.

11. Koonin EV, Fedorova ND, Jackson JD. et al. A comprehensive evolutionary classification of proteins encoded in complete eukaryotic genomes. *Genome biol* 2004;5**:**R7.

12. Huerta-Cepas J, Szklarczyk D, Forslund K. et al. eggNOG 4.5: a hierarchical orthology framework with improved functional annotations for eukaryotic, prokaryotic and viral sequences. *Nucleic Acids Res* 2016;44**:**D286-293.

13. Kanehisa M, Goto S, Kawashima S. et al. The KEGG resource for deciphering the genome. *Nucleic Acids Res* 2004;32**:**D277-280.

14. Buchfink B, Xie C, Huson DH. Fast and sensitive protein alignment using DIAMOND. *Nat Methods* 2015;12**:**59-60.

15. Finn RD, Bateman A, Clements J. et al. Pfam: the protein families database. *Nucleic acids research* 2014;42**:**D222-230.

16. Eddy SR. Profile hidden Markov models. *Bioinformatics (Oxford, England)* 1998;14**:**755-763.

17. Dixon P. VEGAN, a package of R functions for community ecology. *J Veg Sci* 2003;14**:**927-930.

18. Katoh K, Misawa K, Kuma Ki. et al. MAFFT: a novel method for rapid multiple sequence alignment based on fast Fourier transform. *Nucleic Acids Res* 2002;30**:**3059-3066.

19. Price MN, Dehal PS, Arkin AP. FastTree 2 – approximately maximum-likelihood trees for large alignments. *PLoS ONE* 2010;5**:**e9490
