# Supplementary material for: Microbiome-mediated polyphosphate accumulation enhances the resilience of sponge holobionts to future climate scenarios
Source: ISME J. 2026 May 29;20(1):wrag128. doi: 10.1093/ismejo/wrag128 (PMC13431277; doi:10.1093/ismejo/wrag128)
Supplement: Supplementary_material_wrag128 [file supplementary_material_wrag128.zip › Supplementary materials 1_v.docx]

**Microbiome-mediated polyphosphate accumulation enhances the resilience of sponge holobionts to future climate scenarios**

**Supplementary Tables**

Table S1 Primers designed for *vtc4* used for PCR amplification

| Primer name | Primer sequence | PCR-fragments (bp) |
| --- | --- | --- |
| Vtc4-1F | TTYGTNGAAVGRAAGACBCAT | 561 |
| Vtc4-1R | TGCARYTTNACTTCCA |  |
| Vtc4-2F  Vtc4-2R | TTYGTNGAAVGRAAGACBCAT  AAYTTGGARAACTTDGGNAC | 617 |
| Vtc4-3F  Vtc4-3R | ACNGCYTTYCARCTNCC  ATRTCNACRTCCATYTGNGG | 350 |

Table S2 BLAST results of *vtc4* gene

| Number | identity | Coverage | Nearest BLASTP hit | Taxonomy | Accession |
| --- | --- | --- | --- | --- | --- |
| AT-1 | 77.94% | 23% | Agelas oroides genome assembly, chromosome: 1 | *Eukaryota*; *Metazoa*; *Porifera*; *Demospongiae*; *Heteroscleromorpha*; *Agelasida*; *Agelasidae*; *Agelas*. | OX422183.1 |
| AT-2 | 76.47% | 23% | Agelas oroides genomeassembly, chromosome: 2 | *Eukaryota*; *Metazoa*; *Porifera*; *Demospongiae*; *Heteroscleromorpha*; *Agelasida*; *Agelasidae*; *Agelas*. | OX422184.1 |
| AT-3 | 76.47% | 23% | Agelas oroides genomeassembly, chromosome: 2 | *Eukaryota*; *Metazoa*; *Porifera*; *Demospongiae*; *Heteroscleromorpha*; *Agelasida*; *Agelasidae*; *Agelas*. | OX422184.1 |
| AT-4 | 76.47% | 23% | Agelas oroides genomeassembly, chromosome: 2 | *Eukaryota*; *Metazoa*; *Porifera*; *Demospongiae*; *Heteroscleromorpha*; *Agelasida*; *Agelasidae*; *Agelas*. | OX422184.1 |
| AT-6 | 76.47% | 23% | Agelas oroides genomeassembly, chromosome: 2 | *Eukaryota*; *Metazoa*; *Porifera*; *Demospongiae*; *Heteroscleromorpha*; *Agelasida*; *Agelasidae*; *Agelas*. | OX422184.1 |
| AT-8 | 77.94% | 23% | Agelas oroides genomeassembly, chromosome: 1 | *Eukaryota*; *Metazoa*; *Porifera*; *Demospongiae*; *Heteroscleromorpha*; *Agelasida*; *Agelasidae*; *Agelas*. | OX422183.1 |
| AT-10 | 72.99% | 24% | PREDICTED: Xenia sp. Carnegie-2017 | *Eukaryota*; *Metazoa*; *Cnidaria*; *Anthozoa*; *Octocorallia*; *Malacalcyonacea*; *Xeniidae*; *Xenia*. | XM 047001920.1 |

Table S2 The BLAST results of *vtc4* gene (continued)

| Number | identity | Coverage | Nearest BLASTP hit | Taxonomy | Accession |
| --- | --- | --- | --- | --- | --- |
| AT-12 | 48.46% | 83% | uncharacterized protein LOC114949346 [Acropora millepora] | *Eukaryota*;*Opisthokonta*;*Metazoa*;*Eumetazoa*;*Cnidaria*;*Anthozoa*;*Hexacorallia*;*Scleractinia*;*Astrocoeniina*;*Acroporidae*;*Acropora* | XP_029181578.1 |
| AT-13 | 48.94% | 97% | E3 ubiquitin- ligase DZIP3 [Paramuricea clavata] | *Eukaryota*;*Opisthokonta*;*Metazoa*;*Eumetazoa*;*Cnidaria*;*Anthozoa*;*Octocorallia*;*Malacalcyonacea*;*Plexauridae*;*Paramuricea* | CAB4014772.1 |
| AT-14 | 52.36% | 99% | E3 ubiquitin- ligase DZIP3 [Paramuricea clavata] | *Eukaryota*;*Opisthokonta*;*Metazoa*;*Eumetazoa*;*Cnidaria*;*Anthozoa*;*Octocorallia*;*Malacalcyonacea*;*Plexauridae*;*Paramuricea* | CAB4014772.1 |
| AT-15 | 52.36% | 98% | E3 ubiquitin- ligase DZIP3 [Paramuricea clavata] | *Eukaryota*;*Opisthokonta*;*Metazoa*;*Eumetazoa*;*Cnidaria*;*Anthozoa*;*Octocorallia*;*Malacalcyonacea*;*Plexauridae*;*Paramuricea* | CAB4014772.1 |
| AT-16 | 53.38% | 75% | transposase family protein [Nitrosopumilus sp.] | *Archaea*;*Thermoproteati*;*Nitrososphaerota*;*Nitrososphaeria*;*Nitrosopumilales*;*Nitrosopumilaceae*;*Nitrosopumilus*;*unclassified Nitrosopumilus* | MBA4719447.1 |
| AH-1 | 84.52% | 81% | SocA family protein [Ectothiorhodospiraceaebacterium AqS1] | *Bacteria*; *Pseudomonadota*; *Gammaproteobacteria*; *Chromatiales*; *Ectothiorhodospiraceae*. | MBF2761367.1 |
| AH-3 | 84.52% | 81% | SocA family protein [Ectothiorhodospiraceaebacterium AqS1] | *Bacteria*; *Pseudomonadota*; *Gammaproteobacteria*; *Chromatiales*; *Ectothiorhodospiraceae*. | MBF2761367.1 |
| AH-4 | 84.52% | 81% | SocA family protein [Ectothiorhodospiraceaebacterium AqS1] | *Bacteria*; *Pseudomonadota*; *Gammaproteobacteria*; *Chromatiales*; *Ectothiorhodospiraceae*. | MBF2761367.1 |
| AH-5 | 83.87% | 81% | SocA family protein [Ectothiorhodospiraceaebacterium AqS1] | *Bacteria*; *Pseudomonadota*; *Gammaproteobacteria*; *Chromatiales*; *Ectothiorhodospiraceae*. | MBF2761367.1 |
| AH-6 | 42.78% | 97% | PREDICTED: uncharacterizedproteinLOC100637172 [Amphimedonqueenslandica] | *Eukaryota*; *Metazoa*; *Porifera*; *Demospongiae*; *Heteroscleromorpha*; *Haplosclerida*; *Niphatidae*; *Amphimedon*. | XP_011405271.1 |
| AH-7 | 84.52% | 81% | SocA family protein [Ectothiorhodospiraceaebacterium AqS1] | *Bacteria*; *Pseudomonadota*; *Gammaproteobacteria*; *Chromatiales*; *Ectothiorhodospiraceae*. | MBF2761367.1 |

Table S2 The BLAST results of *vtc4* gene (continued)

| Number | Identity | Coverage | | Nearest BLASTP hit | Taxonomy | | Accession |
| --- | --- | --- | --- | --- | --- | --- | --- |
| AH-8 | 83.87% | 81% | SocA family protein [Ectothiorhodospiraceaebacterium AqS1] | | | *Bacteria*; *Pseudomonadota*; *Gammaproteobacteria*; *Chromatiales*; *Ectothiorhodospiraceae*. | MBF2761367.1 |
| AH-9 | 84.52% | 81% | SocA family protein [Ectothiorhodospiraceaebacterium AqS1] | | | *Bacteria*; *Pseudomonadota*; *Gammaproteobacteria*; *Chromatiales*; *Ectothiorhodospiraceae* | MBF2761367.1 |
| AH-13 | 83.87% | 81% | SocA family protein [Ectothiorhodospiraceaebacterium AqS1] | | | *Bacteria*; *Pseudomonadota*; *Gammaproteobacteria*; *Chromatiales*; *Ectothiorhodospiraceae* | MBF2761367.1 |
| AH-14 | 88.44% | 71% | hypothetical protein [Alteromonas sp.] | | | *Bacteria*; *Pseudomonadota*; *Gammaproteobacteria*; *Alteromonadales: Alteromonadaceae*; *Alteromonas/Salinimonas group*; *Alteromonas*. | MCP3704841.1 |
| AH-15 | 83.87% | 81% | SocA family protein [Ectothiorhodospiraceaebacterium AqS1] | | | *Bacteria*; *Pseudomonadota*; *Gammaproteobacteria*; *Chromatiales*; *Ectothiorhodospiraceae*. | MBF2761367.1 |
| AH-16 | 83.87% | 81% | SocA family protein [Ectothiorhodospiraceaebacterium AqS1] | | | *Bacteria*; *Pseudomonadota*; *Gammaproteobacteria*; *Chromatiales*; *Ectothiorhodospiraceae*. | MBF2761367.1 |
| AH-17 | 84.52% | 81% | SocA family protein [Ectothiorhodospiraceaebacterium AqS1] | | | *Bacteria*; *Pseudomonadota*; *Gammaproteobacteria*; *Chromatiales*; *Ectothiorhodospiraceae*. | MBF2761367.1 |
| AH-21 | 87.23% | 64% | DNA polymerase III subunit beta [Luminiphilus sp.] | | | *Bacteria*; *Pseudomonadati*; *Pseudomonadota*; *Gammaproteobacteria*; *Cellvibrionales*; *Halieaceae*; *Luminiphilus* | MBL6697954.1 |
| AH-22 | 84.52% | 81% | SocA family protein [Ectothiorhodospiraceaebacterium AqS1] | | | *Bacteria*; *Pseudomonadota*; *Gammaproteobacteria*; *Chromatiales*; *Ectothiorhodospiraceae* | MBF2761367.1 |

Table S2 The BLAST results of *vtc4* gene (continued)

| Number | identity | Coverage | Nearest BLASTP hit | Taxonomy | Accession |
| --- | --- | --- | --- | --- | --- |
| AH-23 | 84.52% | 81% | SocA family protein [Ectothiorhodospiraceaebacterium AqS1] | *Bacteria*; *Pseudomonadota*; *Gammaproteobacteria*; *Chromatiales*; *Ectothiorhodospiraceae* | MBF2761367.1 |
| AH-24 | 83.87% | 81% | SocA family protein [Ectothiorhodospiraceaebacterium AqS1] | *Bacteria*; *Pseudomonadota*; *Gammaproteobacteria*; *Chromatiales*; *Ectothiorhodospiraceae* | MBF2761367.1 |
| AH-25 | 83.87% | 81% | SocA family protein [Ectothiorhodospiraceaebacterium AqS1] | *Bacteria*; *Pseudomonadota*; *Gammaproteobacteria*; *Chromatiales*; *Ectothiorhodospiraceae* | MBF2761367.1 |
| AH-26 | 84.52% | 81% | SocA family protein [Ectothiorhodospiraceaebacterium AqS1] | *Bacteria*; *Pseudomonadota*; *Gammaproteobacteria*; *Chromatiales*; *Ectothiorhodospiraceae* | MBF2761367.1 |
| AH-27 | 84.52% | 81% | SocA family protein [Ectothiorhodospiraceaebacterium AqS1] | *Bacteria*; *Pseudomonadota*; *Gammaproteobacteria*; *Chromatiales*; *Ectothiorhodospiraceae* | MBF2761367.1 |
| BH-2 | 89.04% | 70% | hypothetical protein [Alteromonas sp.] | *Bacteria*; *Pseudomonadota*; *Gammaproteobacteria*; *Alteromonadales*; *Alteromonadaceae*; *Alteromonas/Salinimonas group*; *Alteromonas* | MCP3704841.1 |
| BH-3 | 89.12% | 72.00% | hypothetical protein [Alteromonas sp.] | *Bacteria*; *Pseudomonadota*; *Gammaproteobacteria*; *Alteromonadales*; *Alteromonadaceae*; *Alteromonas/Salinimonas group*; *Alteromonas* | MCP3704841.1 |
| BH-4 | 97.74% | 60.00% | nucleotidyltransferase family proteinParacoccaceae bacterium | *Bacteria*; *Pseudomonadota*; *Alphaproteobacteria*; *Rhodobacterales*; *Paracoccaceae* | MBR9769293.1 |
| BH-5 | 68.41% | 77.00% | Ruegeria sp.TM1040 plasmid megaplasmidcomplete sequence | *Bacteria*; *Pseudomonadota*; *Alphaproteobacteria*; *Rhodobacterales*; *Roseobacteraceae*; *Ruegeria* | CP000376.1 |

Table S2 The BLAST results of *vtc4* gene (continued)

| Number | identity | Coverage | Nearest BLASTP hit | Taxonomy | Accession |
| --- | --- | --- | --- | --- | --- |
| BH-6 | 68.41% | 77.00% | Ruegeria sp.TM1040 plasmid megaplasmidcomplete sequence | *Bacteria*; *Pseudomonadota*; *Alphaproteobacteria*; *Rhodobacterales*; *Roseobacteraceae*; *Ruegeria* | CP000376.1 |
| BH-8 | 89.12% | 72.00% | hypothetical protein [Alteromonas sp.] | *Bacteria*; *Pseudomonadota*; *Gammaproteobacteria*; *Alteromonadales: Alteromonadaceae*; *Alteromonas/Salinimonas group*; *Alteromonas* | MCP3704841.1 |
| BH-9 | 89.12% | 71% | hypothetical protein [Alteromonas sp.] | *Bacteria*; *Pseudomonadota*; *Gammaproteobacteria*; *Alteromonadales: Alteromonadaceae*; *Alteromonas/Salinimonas group*; *Alteromonas* | MCP3704841.1 |
| BH-10 | 88.44% | 71% | hypothetical protein [Alteromonas sp.] | *Bacteria*; *Pseudomonadota*; *Gammaproteobacteria*; *Alteromonadales: Alteromonadaceae*; *Alteromonas/Salinimonas group*; *Alteromonas* | MCP3704841.1 |
| BH-11 | 55.56% | 76% | Gfo/Idh/MocA family oxidoreductase [Ectothiorhodospiraceae bacterium AqS1] | *Bacteria*; *Pseudomonadota*; *Gammaproteobacteria*; *Chromatiales*; *Ectothiorhodospiraceae* | MBF2759535.1 |
| BH-12 | 89.04% | 71% | hypothetical protein [Alteromonas sp.] | *Bacteria*; *Pseudomonadota*; *Gammaproteobacteria*; *Alteromonadales: Alteromonadaceae*; *Alteromonas/Salinimonas group*; *Alteromonas* | MCP3704841.1 |
| BH-13 | 88.36% | 70% | hypothetical protein [Alteromonas sp.] | *Bacteria*; *Pseudomonadota*; *Gammaproteobacteria*; *Alteromonadales: Alteromonadaceae*; *Alteromonas/Salinimonas group*; *Alteromonas* | MCP3704841.1 |

Table S2 The BLAST results of *vtc4* gene (continued)

| Number | identity | Coverage | Nearest BLASTP hit | Taxonomy | Accession |
| --- | --- | --- | --- | --- | --- |
| BH-14 | 88.44% | 71% | hypothetical protein [Alteromonas sp.] | *Bacteria*; *Pseudomonadota*; *Gammaproteobacteria*; *Alteromonadales: Alteromonadaceae*; *Alteromonas/*Salinimonas group; *Alteromonas* | MCP3704841.1 |
| BH-15 | 55.56% | 76% | Gfo/Idh/MocA family oxidoreductase [Ectothiorhodospiraceae bacterium AqS1] | *Bacteria*; *Pseudomonadota*; *Gammaproteobacteria*; *Chromatiales*; *Ectothiorhodospiraceae* | MBF2759535.1 |
| BH-16 | 88.44% | 71% | hypothetical protein [Alteromonas sp.] | *Bacteria*; *Pseudomonadota*; *Gammaproteobacteria*; *Alteromonadales: Alteromonadaceae*; *Alteromonas/*Salinimonas group; *Alteromonas* | MCP3704841.1 |
| BH-17 | 55.56% | 76% | Gfo/Idh/MocA family oxidoreductase [Ectothiorhodospiraceae bacterium AqS1] | *Bacteria*; *Pseudomonadota*; *Gammaproteobacteria*; *Chromatiales*; *Ectothiorhodospiraceae* | MBF2759535.1 |
| BH-18 | 88.44% | 71% | hypothetical protein [Alteromonas sp.] | *Bacteria*; *Pseudomonadota*; *Gammaproteobacteria*; *Alteromonadales: Alteromonadaceae*; *Alteromonas/*Salinimonas group; *Alteromonas* | MCP3704841.1 |

AT and AH were BLAST results for primer pairs of Vtc4-1F and Vtc4-1R. BH were BLAST results for primer pairs of Vtc4-2F and Vtc4-2R. T = *Tedania* sp., H = *Haliclona simulans*

**Supplementary Figures**


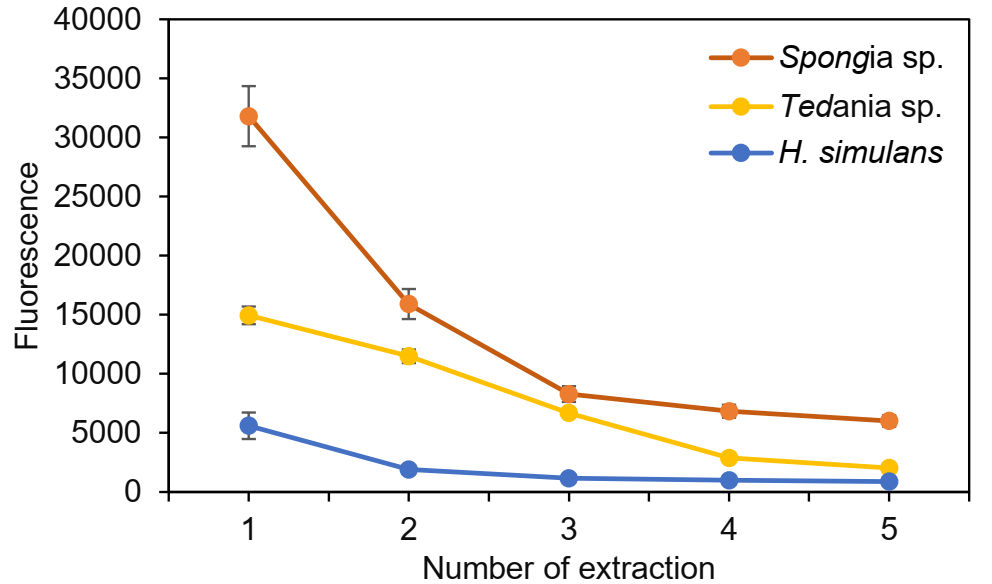


Fig. S1 Fluorescence reading from five successive PolyP extractions from lyophilized samples of three sponge species. Stained with DAPI in triplicate. Error bars indicate ±1 SD.


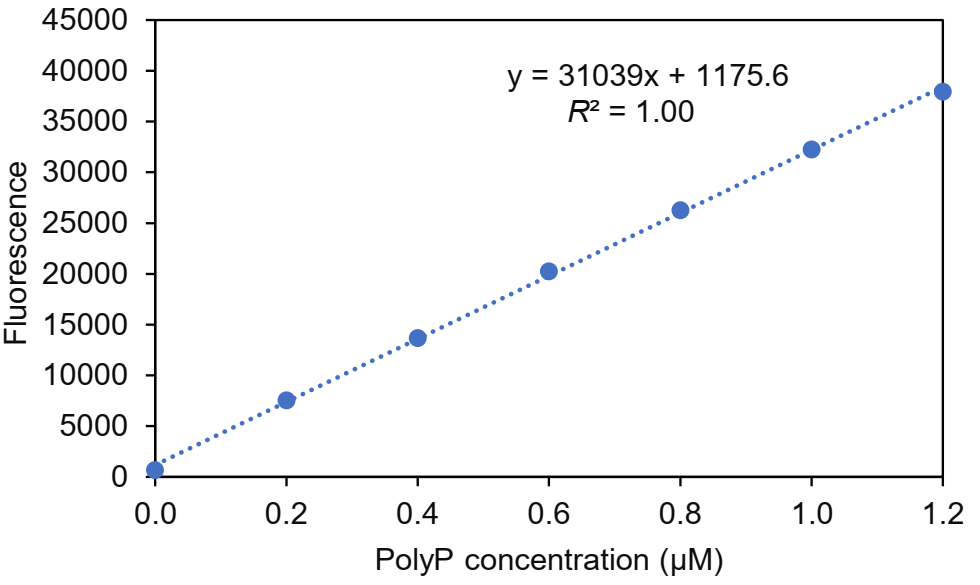


Fig. S2 Standard curve of the concentration of PolyP.


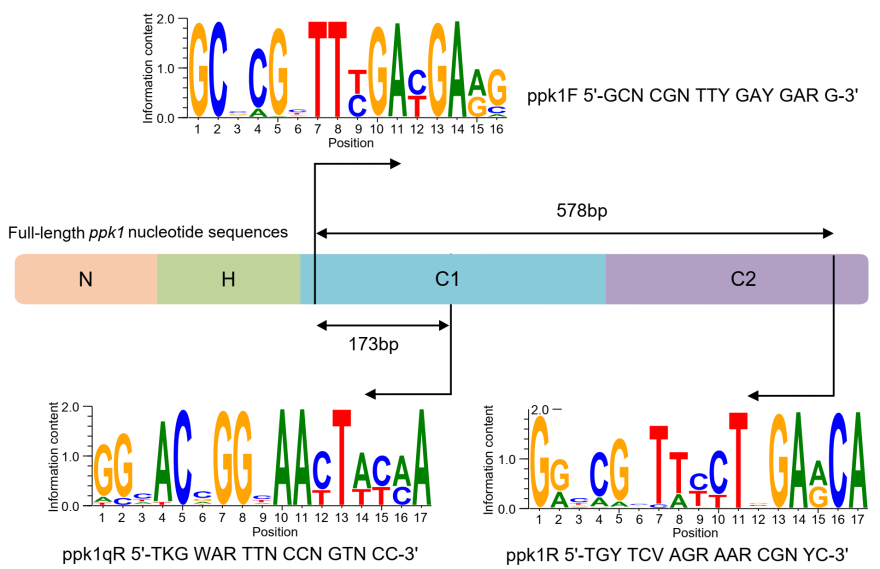


Fig. S3 Primers for PCR and qRT-PCR are designed based on the full-length *ppk1* nucleotide sequence. Polyphosphate kinase (PPK), which contains four structural domains per chain: N-terminal (N) domain, head (H) domain, and two closely related carboxy-terminal (C1 and C2) domains.Arrows indicate primer binding sites and amplicon size.


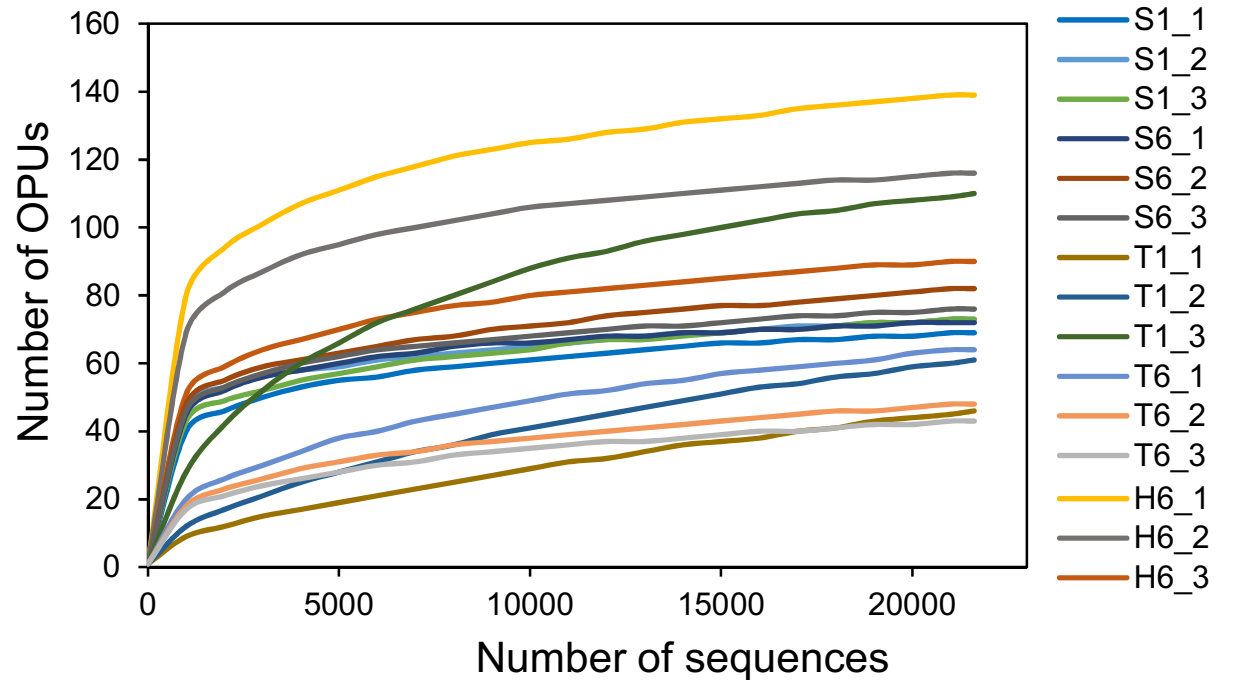


Fig. S4 Rarefaction curves of operational protein units (OPUs). S = *Spongia* sp., T = *Tedania* sp., H = *Haliclona simulans*. Arabic numerals after the letters represent months.


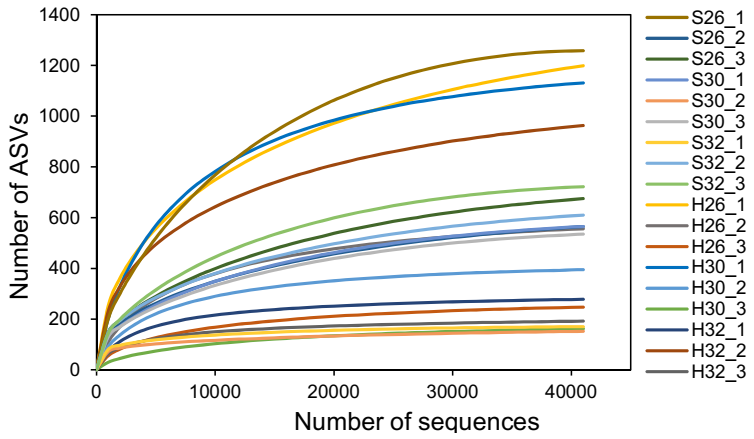


Fig. S5 Rarefaction curves of amplicon sequence variants (ASVs). S = *Spongia* sp., H = *Haliclona simulans*. The Arabic numerals following the letters indicate the temperature at which the sponge was exposed.


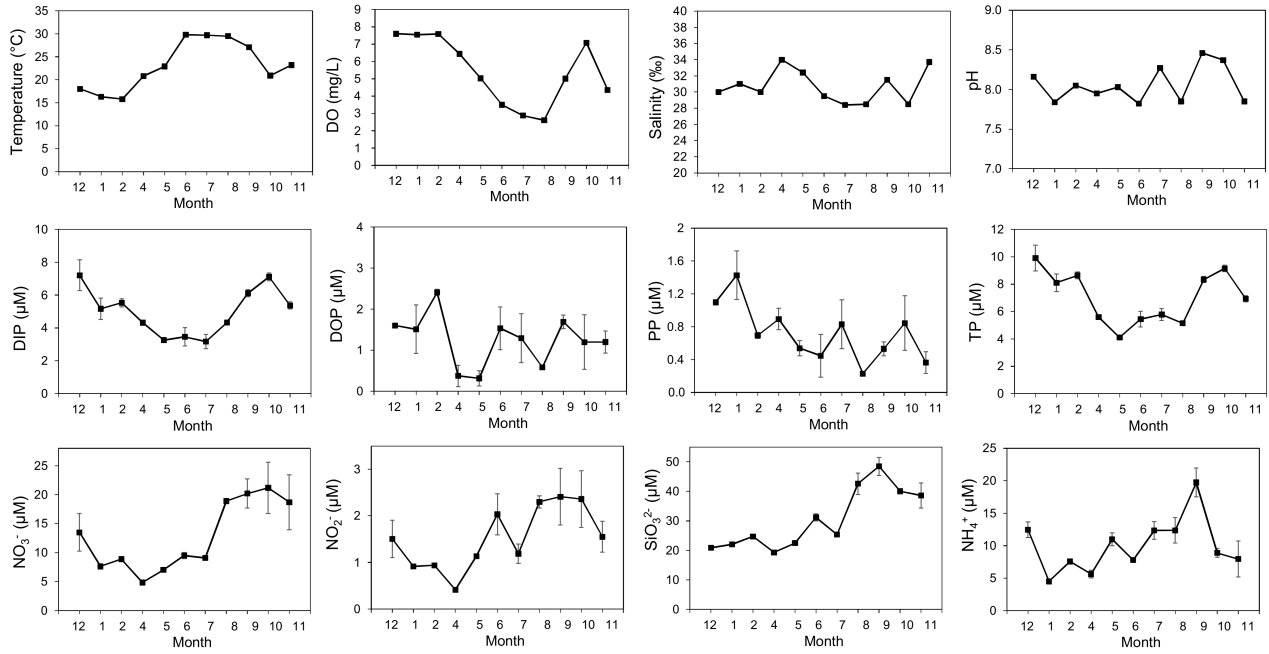


Fig. S6 Physicochemical parameters of seawater during sampling. DOP was calculated as the difference between the mean concentrations of TDP and DIP.


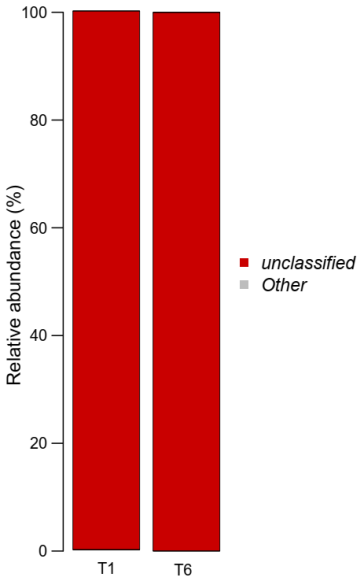


Fig. S7 *vtc4*-harbing microbial community composition at phylum level. The primer pairs of Vtc4-3F and Vtc4-3R. T = *Tedania* sp.


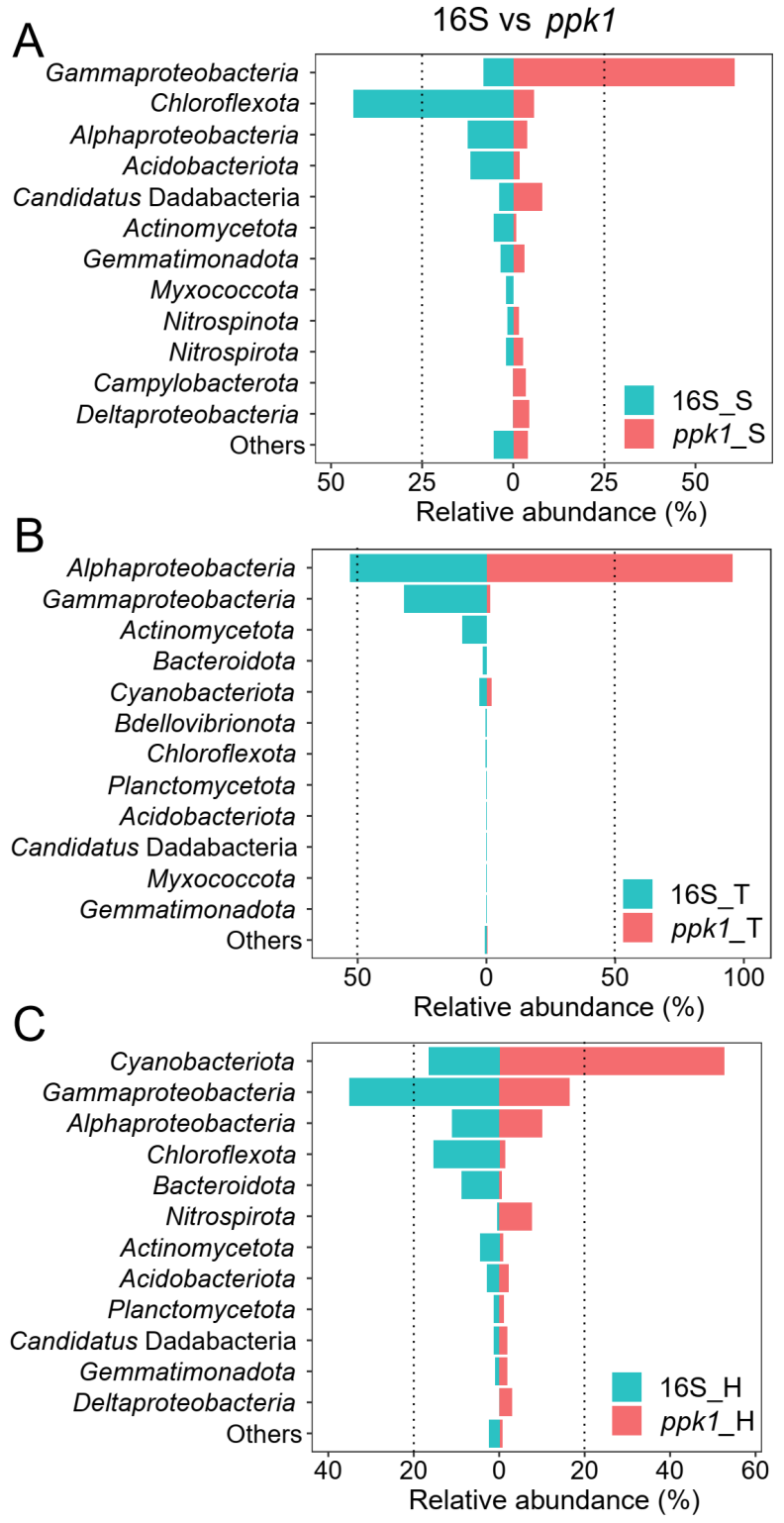


Fig. S8 Comparison of the relative abundance of *ppk1*-harboring microbial assemblages and sponge-associated microbes in *Spongia* sp. (A), *Tedania* sp. (B), and *H*. *simulans* (C)*.* S = *Spongia* sp., T = *Tedania* sp., H = *H*. *simulans*.


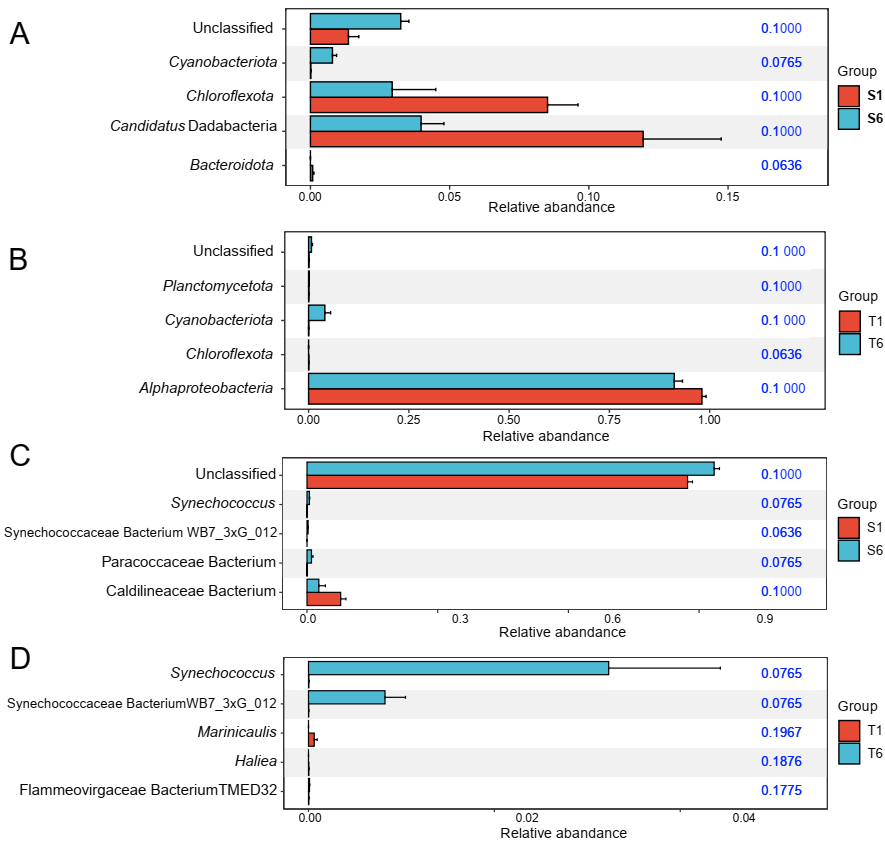


Fig. S9 Wilcoxon test of the relative abundance of *ppk1*-harboring microbial phyla (A, B) and genera (C, D) in sponges in January and June. S = *Spongia* sp., T = *Tedania* sp.


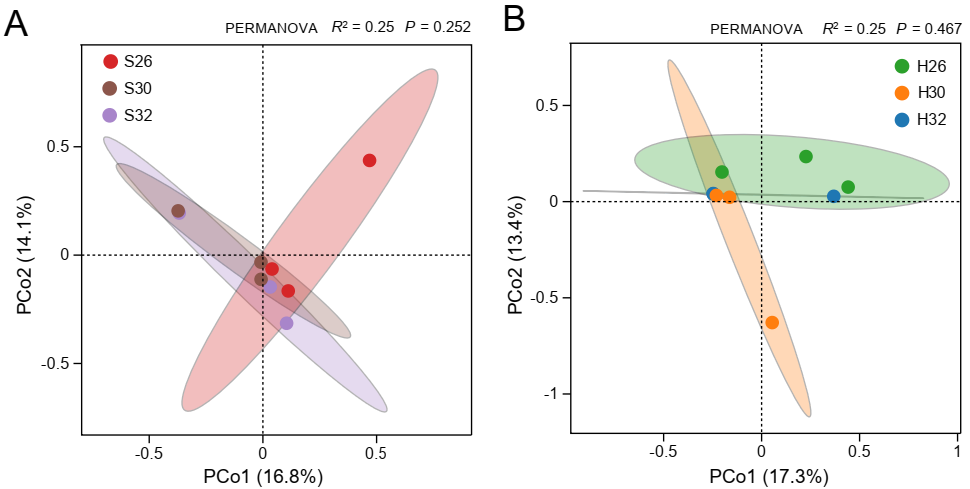


Fig. S10 Bacterial community structure of *Spongia* sp. (A) and *H*. *simulans* (B). Principal Coordinate Analysis (PCoA) based on Bray–Curtis dissimilarity matrix. S = *Spongia* sp., H = *H*. *simulans*. Numbers indicate the exposure temperature.


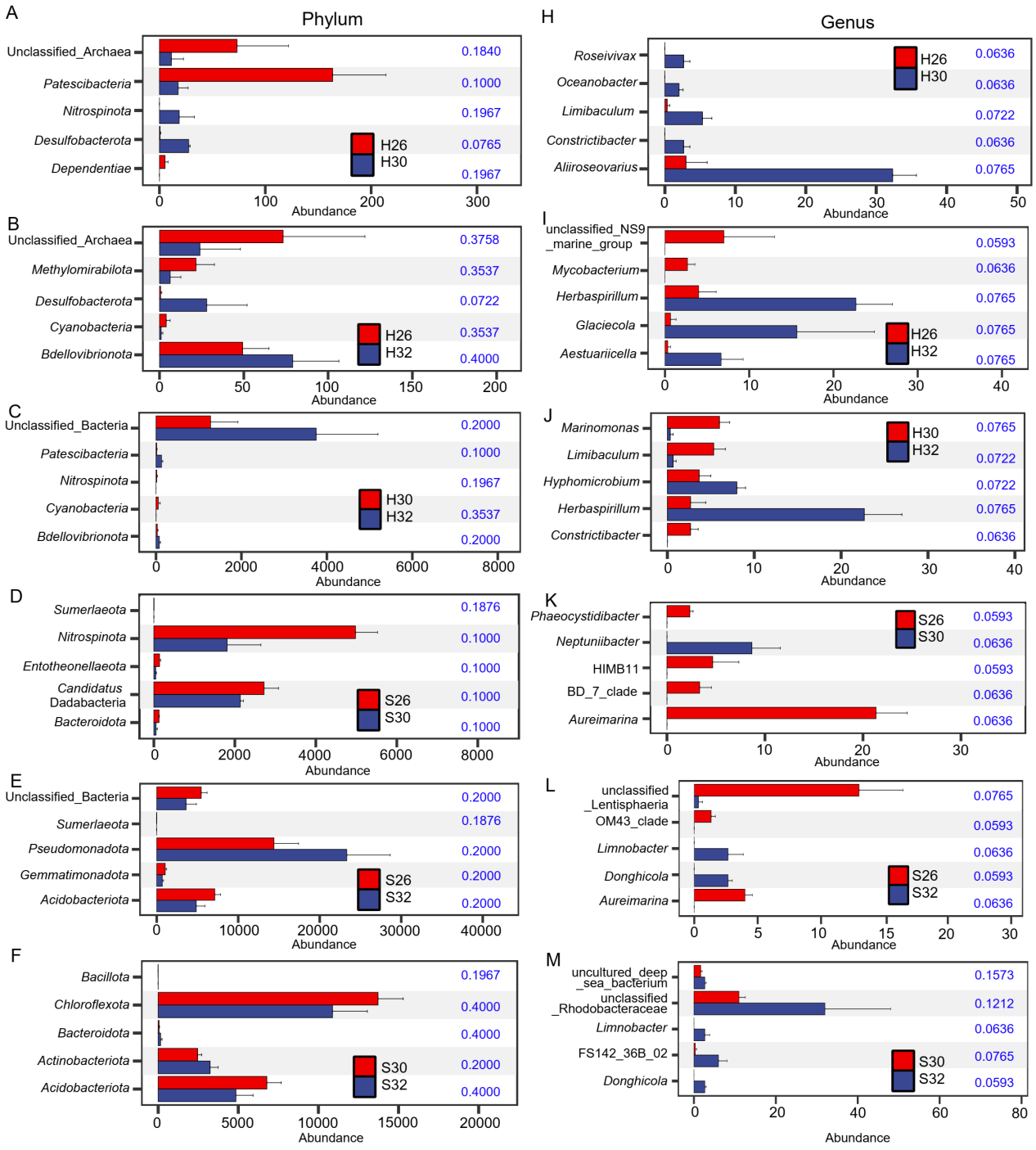


Fig. S11 Wilcoxon test of the relative abundance of bacterial phyla (A-F) and genera (H-M) in sponges. S = *Spongia* sp., T = *Tedania* sp. Numbers indicate the exposure temperature.


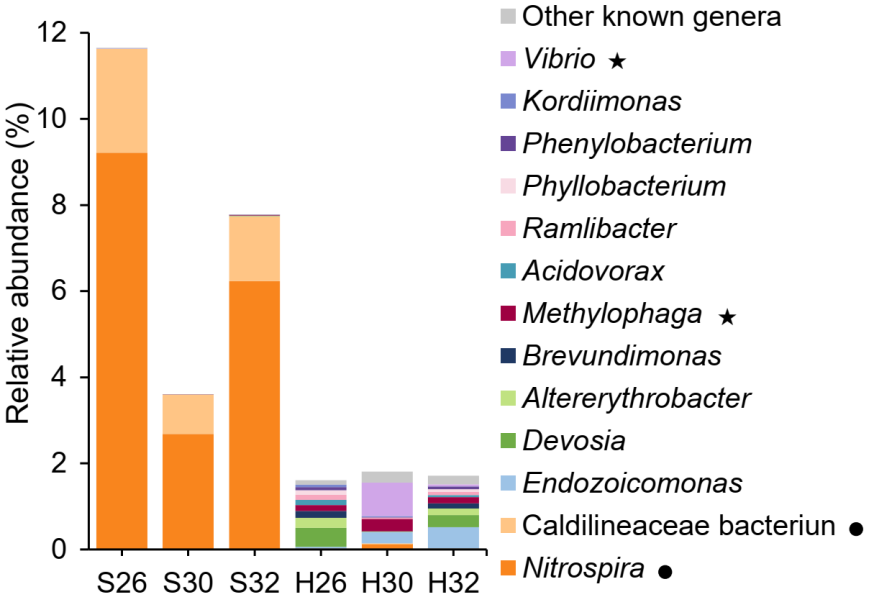


Fig. S12 Relative abundance of *ppk1*-harboring microbial genera. ★: The relative abundance of microorganisms increased first and then decreased under various temperature regimes. ●: The relative abundance of microorganisms decreased first and then increased. S = *Spongia* sp., H = *H*. *simulans*.


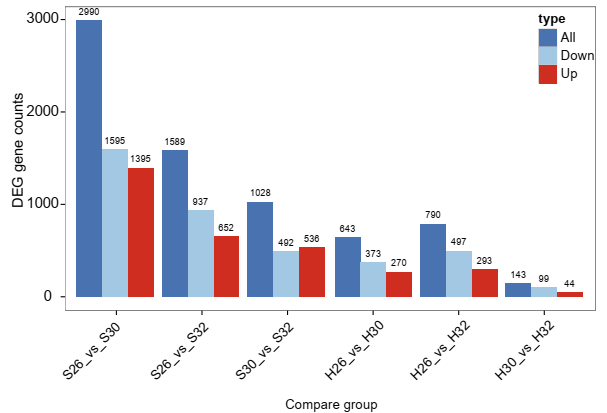


Fig. S13 Number of differentially expressed genes (DEGs) in *Spongia* sp. and *H*. *simulans* under various temperature regimes.


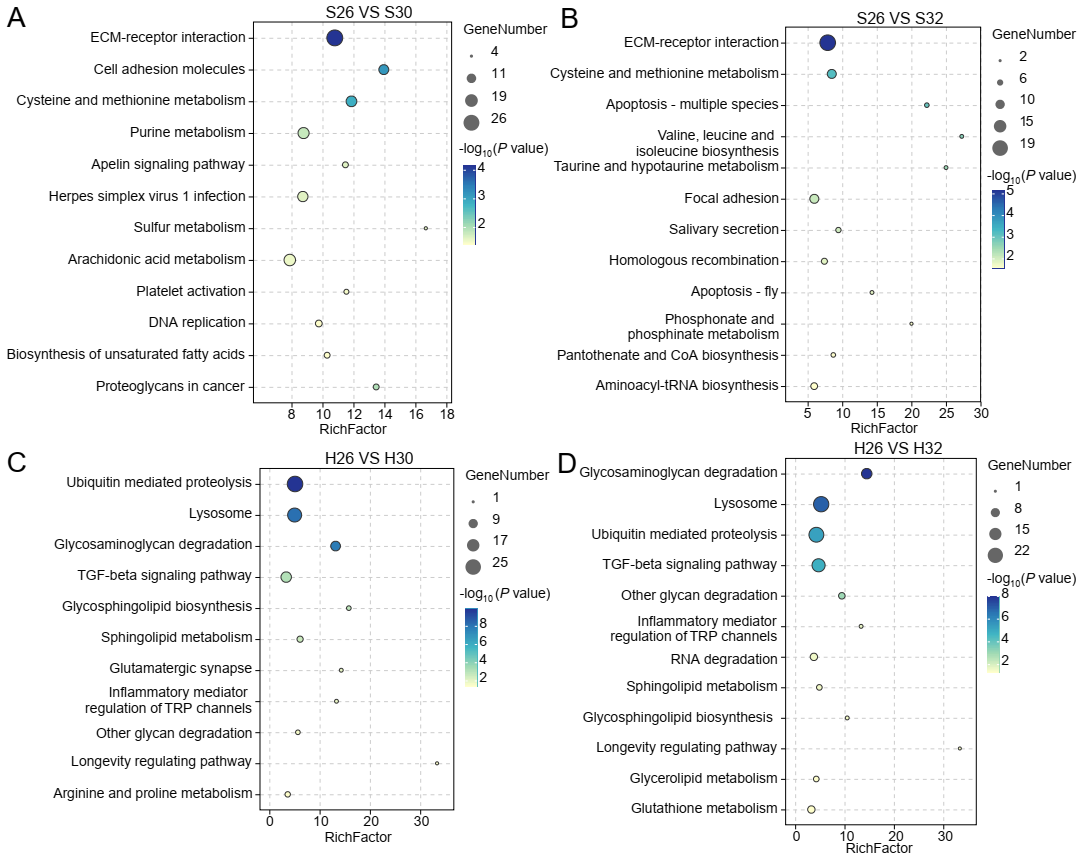


Fig. S14 Kyoto Encyclopaedia of Genes and Genomes (KEGG) enrichment analysis for transcripts in *Spongia* sp. (A, B) and *H*. *simulans* (C, D) under various temperature regimes. S = *Spongia* sp., H = *H*. *simulans*. Numbers indicate the exposure temperature.


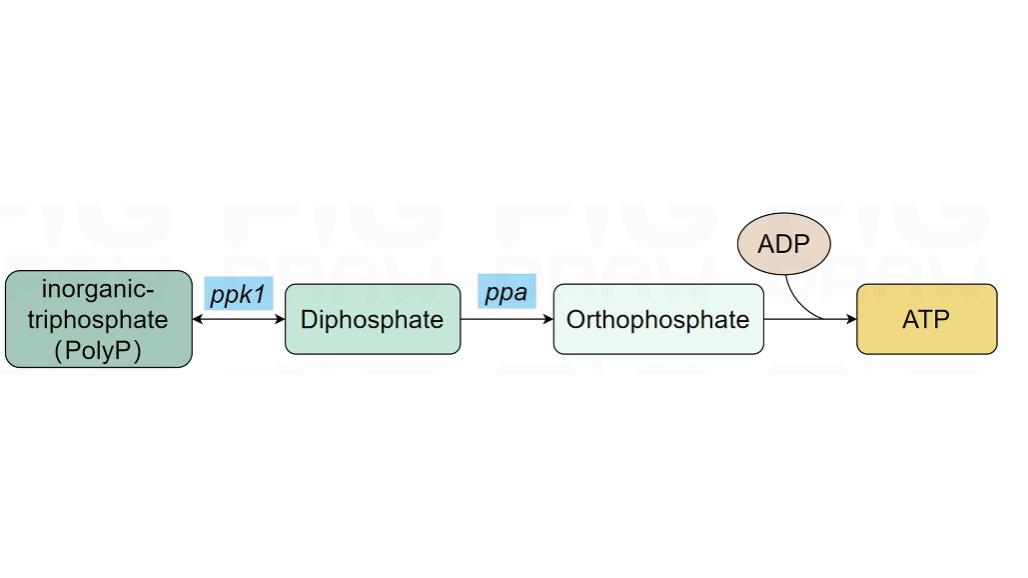


Fig. S15 Simplified diagram of oxidative phosphorylation process.
